# The Discriminative Ability of ROTEM for Delayed Cerebral Ischemia and Poor Outcome Following Aneurysmal Subarachnoid Hemorrhage

**DOI:** 10.1007/s12028-025-02309-x

**Published:** 2025-07-16

**Authors:** M. A. Tjerkstra, H. Labib, B. A. Coert, R. Post, W. P. Vandertop, D. Verbaan, N. P. Juffermans

**Affiliations:** 1 Department of Neurosurgery, Amsterdam University Medical Center, University of Amsterdam, Meibergdreef 9, Amsterdam, the Netherlands; 2 Laboratory of Experimental Intensive Care and Anesthesiology, Amsterdam University Medical Center, University of Amsterdam, Meibergdreef 9, 1105 AZ Amsterdam, the Netherlands; 3 Department of Intensive Care, Erasmus University Medical Center, Dr. Molewaterplein 40, 3015 GD Rotterdam, the Netherlands

**Keywords:** Subarachnoid hemorrhage, Coagulation, Delayed cerebral ischemia, Rotational thromboelastometry, Prognostication, Outcome

## Abstract

**Background:**

The prediction of delayed cerebral ischemia (DCI) and poor clinical outcome following aneurysmal subarachnoid hemorrhage (aSAH) is an unmet clinical need to improve on stratification of patients. DCI and poor clinical outcome following aSAH have been associated with hypercoagulability as detected by viscoelastic testing. This study assesses temporal alterations in rotational thromboelastography (ROTEM) coagulation profiles and the discriminative ability of ROTEM parameters for DCI and poor clinical outcome following aSAH.

**Methods:**

ROTEM parameters were measured on admission, days 3–5, and days 9–11 after aSAH and compared between patients with and without DCI, radiological DCI, and poor 6-month clinical outcome as per modified Rankin Scale scores 4–6. Receiver operating characteristic curve analyses were used to calculate areas under the curve (AUCs) and determine cutoff values with a sensitivity > 90% for (radiological) DCI and with a specificity > 90% for poor outcome.

**Results:**

Of 160 included patients with aSAH, 31 (19%) had DCI, 16 (10%) had radiological DCI, and 68 (44%) had poor outcome at 6 months. DCI, radiological DCI, and poor clinical outcome were associated with hypercoagulability. The ROTEM parameter with the best discriminative ability for radiological DCI was INTEM clotting time (AUC 0.75) on admission day, with an optimal cutoff value of < 146 s (sensitivity 92%, specificity 47%). For poor outcome, this was increased clot strength by FIBTEM amplitude at 10 minutes (A10, AUC 0.85) on days 3–5, with an optimal cutoff value > 27 mm (specificity 94%, sensitivity 49%).

**Conclusions:**

In this study, ROTEM parameters indicative of increased coagulation had good predictive ability for poor clinical outcome. If independently validated, ROTEM parameters might have the potential to stratify patients with aSAH who may benefit from anticoagulant treatment in future trials with the aim to improve clinical outcome.

**Supplementary Information:**

The online version contains supplementary material available at 10.1007/s12028-025-02309-x.

## Introduction

Aneurysmal subarachnoid hemorrhage (aSAH) has a case fatality rate of 32–42% and a permanent disability rate of 50% among survivors [1]. In patients who survive the first 24 h, the most important contributor to poor outcome is delayed cerebral ischemia (DCI), occurring between days 3 and 12 post-SAH in one of three to four patients with aSAH [2–5]. Other complications include rebleeding, hydrocephalus, or nosocomial infection, which collectively contribute to adverse outcome. To monitor these complications, patients with aSAH are hospitalized for at least 14 days. The pathophysiology of DCI is thought to be multifactorial, including neuroinflammation and microthrombi formation [6, 7]. However, although anticoagulant therapy reduces occurrence of secondary ischemia, this benefit is offset by an increase in bleeding risk, suggesting that some patients may benefit from anticoagulation, but others clearly do not. Thereby, no recommendation on anticoagulant management is given in guidelines [8–10]. These results highlight the need for improved recognition of those patients likely to benefit from anticoagulant strategies.

Thus far, individual markers of coagulation and fibrinolysis have shown no predictive association with DCI [11]. Rotational thromboelastometry (ROTEM) analyzes whole blood and provides information about the contribution of coagulation factors, platelet function, and fibrinolysis [12]. Studies on ROTEM have shown increased coagulation in patients with aSAH compared to controls [13]. These studies are limited by small sample sizes, hampering determination of specific cutoff values.

The aim of this study is to evaluate the ability of ROTEM to predict complications in patients with aSAH by determining specific cutoff values for DCI and poor clinical outcome at 6 months. We hypothesize that patients with aSAH with DCI and those with poor clinical outcome have a hypercoagulable ROTEM profile when compared to patients without DCI or those with good clinical outcome.

## Methods

### Study Design

A prospective cohort study was performed from October 2018 to May 2023 at the Amsterdam University Medical Center. The study was interrupted during the COVID-pandemic. Amsterdam University Medical Center is a tertiary referral center for patients with aSAH. Consecutive patients were included if they met the following criteria: (1) aged 18 or older, (2) diagnosed with a nontraumatic SAH by either subarachnoid blood on noncontrast head computed tomography or a positive lumbar puncture result, and (3) had blood sample collected within 24 h after ictus and before aneurysm treatment. Patients with nonaneurysmal SAH were excluded. The study was conducted in accordance with the Declaration of Helsinki. The protocol was approved by our institutional review board (MEC 2017_318). Deferred informed consent was obtained from patients or their legal representatives, except for those who suffered imminent death. Standard of care comprised securing the aneurysm < 72 h by endovascular coiling or surgical clipping. All patients received nadroparine (patients < 100 kg: 2850 IU; patients > 100 kg: 5700 IU) as thrombosis prophylaxis starting on day 0 following aneurysm treatment. Patients received antiplatelet therapy if intraarterial thrombosis occurred during endovascular treatment (daily acetylsalicylic acid for 3 months) or if treatment included stent-assisted coiling (daily clopidogrel and acetylsalicylic acid) or a flow diverter (prasugrel) for at least 6 months. During the first part of the inclusion period, a trial on tranexamic acid treatment was conducted in the same patient group [14].

### Data Collections

The following variables were collected: baseline characteristics (including the World Federation of Neurological Surgeons [WFNS] grade and Fisher grade), common complications following aSAH (definitions in Supplemental Files, page 1), laboratory values, use of hemostatic agents during hospitalization, and clinical outcome assessed by the modified Rankin Scale (mRS) at 6 months after aSAH, which was scored by a trained nurse using a structured and validated interview at the outpatient clinic or by telephone.

### Blood Sampling

Citrated BD Vacutainer tubes were collected at onset of symptoms (day 0; T0) and on days 3–5 after ictus (T1). For the first 98 patients, we also collected blood samples at days 9–11 after aSAH (T2). Due to limited funding and based on preliminary results (data not published) the T2 sample collection was stopped after the first 98 patients. Blood samples were used for ROTEM analysis within 2 h after withdrawal.

### Coagulation Tests

**Fig. 1:**
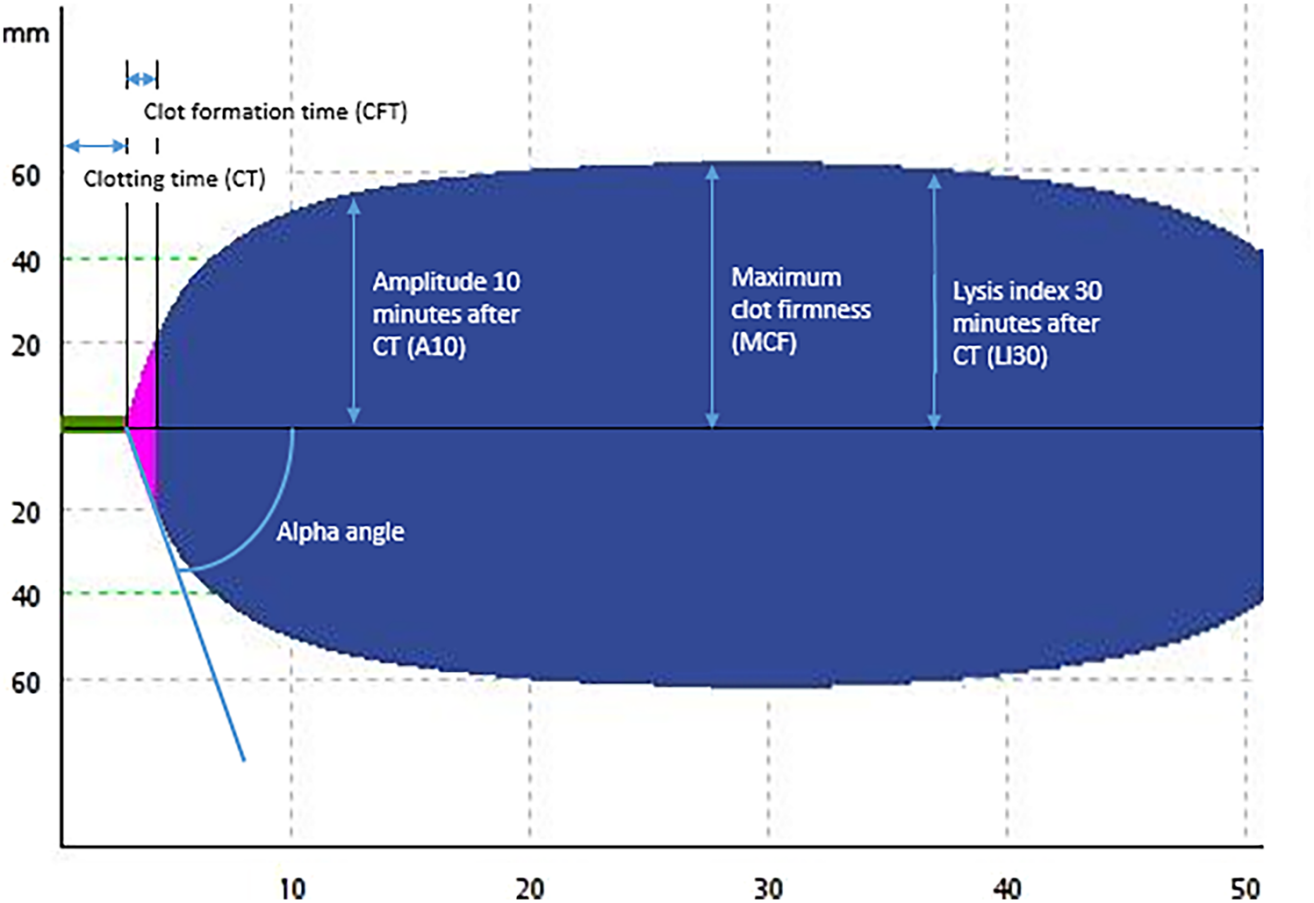
Depiction of rotational thromboelastometry (ROTEM) parameters

Using ROTEM Sigma (Werfen, Benelux), a citrated blood sample was inserted into a cartridge containing INTEM, EXTEM, and FIBTEM assays. Of the EXTEM and INTEM assays, we used clotting time (CT), clot formation time (CFT), α-angle, amplitude at 10 min (A10), maximum clot firmness (MCF), and clot lysis index at 60 min. Of the FIBTEM assays, we used a-angle, A10, and MCF. CT depicts the time until clot initiation. CFT and α-angle depict the speed of clot formation. A10 and MCF depict the clot strength at 10 min after clotting time and the maximum clot strength (Fig. 1).

Conventional coagulation parameters were measured with Sysmex CS-2500 and included prothrombin time (PT), activated partial thromboplastin time (aPTT), fibrinogen level, and D-dimer level. Stored citrate plasma samples were thawed in a 37-°C water bath and vortexed. The following reagents were used: Innovin PT reagents (Siemens B4212–100), Actin FS (Siemens B4218–100), and 25 mmol/L CaCl_2_ (Siemens ORHO375) for aPTT; Thrombin Reagent (Siemens B4233–25) for fibrinogen; and Innovance D-Dimer (Siemens OPBO07) for D-dimer. The detection limits were 7.0 to 180.0 s for PT, 20.0 to 180.0 s for aPTT, 0.3 to 8.9 g/L for fibrinogen, and 0.2 to 35.2 mg/L for D-dimer. If a measurement exceeded the detection limit, the value was set at the detection limit. The results of ROTEM and standard coagulation tests did not influence the management of patients with aSAH.

### Outcome Measurements

During the first 14 days of admission, patients were evaluated every 4 to 8 h for neurologic deterioration. If new focal neurological deficits or a decrease in consciousness occurred lasting for at least 1 h, patients underwent clinical assessment, clinical imaging, laboratory testing, and, in some cases, electroencephalography to rule out epileptiform activity. If no other explanatory cause was found for the neurological decline, the patient was classified as having clinical DCI [15]. Radiological DCI was defined as the presence of cerebral infarction on CT or magnetic resonance imaging (MRI) (irrespective of the presence of clinical symptoms of DCI) that was not present on computed tomography or MRI within 48 h after aSAH and cannot be attributed to the aneurysm treatment or other causes [15]. Radiological DCI was scored based on all available computed tomography or MRI data during the admission of patients with aSAH. Computed tomography or MRI was only performed on clinical indication. Poor clinical outcome was defined as an mRS score of 4–6 at 6 months.

### Management of aSAH and DCI

Patients were treated according to our standardized protocol, which was mainly based on the International Guidelines of 2013. All patients received thrombotic prophylaxis using nadroparin once daily (patients < 100 kg: 2,850 IU; > 100 kg: 5,700 IU), starting on day 0 and after the first blood draw. Ruptured aneurysms were treated as early as feasible by either neurosurgical clipping or endovascular treatment. When an intraarterial thrombosis occurred during endovascular treatment, lysis of the thrombus was attempted by intraarterial injection of abciximab (Reopro), followed by 100 mg of acetylsalicylic acid for 3 months. If the configuration of the ruptured aneurysm required stent-assisted coiling or a flow diverter, the procedure was preferably postponed for at least 10 days. Afterward, patients were treated with (dual) antiplatelet therapy. In case of hydrocephalus or suspected intracranial hypertension, cerebrospinal fluid was drained either by (repeated) lumbar punctures or via continuous lumbar or external ventricular drainage. Preventive treatment of DCI included calcium antagonists (60 mg of oral nimodipine six times daily) and maintaining normovolemia. When DCI was clinically suspected, patients were treated with induced hypertension.

### Statistical Analyses

At the time our study protocol was accepted in 2018, no studies on ROTEM and DCI had been published, which precluded us from performing a sample size calculation. Baseline characteristics were provided as means with standard deviation (SD), medians with interquartile range (IQR), or proportions with percentages, depending on the type and distribution of data. Normality of continuous variables was tested by the Shapiro–Wilk test (threshold for normality: W > 0.9). To evaluate the temporal alterations in coagulation profiles, group differences were assessed on all three time points between patients with and without complications, using either the independent *t*-test or the Mann–Whitney *U*-test. Given that the different read-out parameters of ROTEM have strong interdependency (see Fig. 1), correction for multiple testing was not deemed adequate, similar to previous studies on the utility of ROTEM to monitor bleeding [16]. Statistical significance was set at *p* < 0.05. Additionally, a sensitivity analysis was done after exclusion of patients who received tranexamic acid in the context of the ULTRA-trial [14].

Parameters with significant differences between groups were selected for calculation of odds ratios with corresponding 95% confidence intervals (95% CIs) by univariate logistic regression. Only ROTEM parameters obtained prior to DCI onset were included in the analysis. ROTEM parameters that were significantly associated with DCI in univariate logistic regression analyses were used to calculate the area under the curve (AUC) with the corresponding 95% CI. An AUC of 0.5 suggests no discriminative ability, an AUC of 0.7–0.8 is considered acceptable, an AUC of 0.8–0.9 is considered excellent, and an AUC > 0.9 is considered outstanding [17]. For the ROTEM parameter with the highest AUC, the sensitivity and specificity were calculated to determine an optimal cutoff value [18–20]. For DCI, it was considered of interest to identify those with high certainty to develop the complication (high proportion of true positive). For poor outcome, it was of interest to identify patients who would not have poor outcome with a high level of certainty (low proportion of false negative).

## Results

**Fig. 2:**
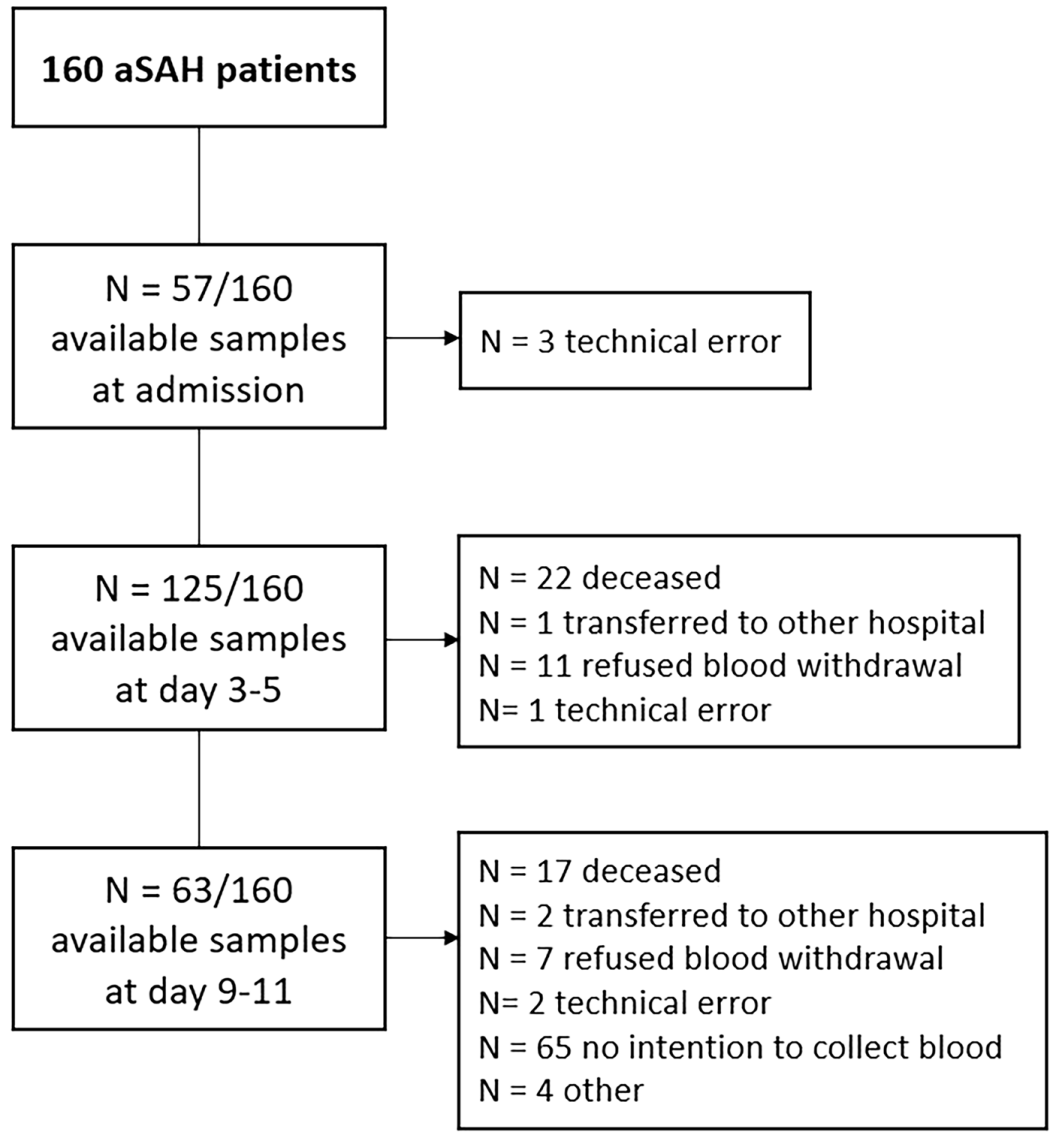
Available blood samples on admission, days 3–5, and days 9–11 after ictus of 160 patients with aneurysmal subarachnoid hemorrhage (aSAH)

The cohort consisted of 160 patients with aSAH, with a mean age of 57.8 years (SD 12.5). The majority were female (*n* = 114, 71%) and had a Fisher grade 3–4 hemorrhage (*n* = 156, 97%) on initial computed tomography. Seventy-nine (50%) presented with WFNS grade 4–5, and 156 (97%) patients had Fisher grade 3–4. The ruptured aneurysm was treated surgically in 14 (9%) patients and endovascularly in 124 (78%) patients. Stent-assisted coiling was performed in three patients (2%), and a flow diverter was placed in two patients (1%). Twenty-two patients did not have their aneurysm treated (moribund condition on admission: 16 of 22; aneurysm deemed technically unsuitable for aneurysm treatment: 6 of 22). Clinical DCI occurred in 31 patients (19%) at a median of 9 days (range 2–16) after aSAH. Radiological DCI was seen in 16 (10%) patients, and 68 (44%) patients had poor outcome. ROTEM measurements were available for 157 of 160 patients (98%) on admission, 125 of 160 (78%) patients on days 3–5, and 63 of 160 (39%) patients on day 10 after aSAH. Reasons for missing ROTEM measurements are listed in Fig. 2. Twenty-six patients with aSAH received ultra-early and short-term (< 24 h) tranexamic acid as participants of the ULTRA-trial (comparison of ROTEM parameters in patients with and without tranexamic acid treatment in Supplemental Files, Table S1, page 2). None of the baseline characteristics and common complications following aSAH, except for treatment modality (*p* = 0.03), significantly differed between patients with and without DCI (Table 1).

**Table 1:** Baseline characteristics and common complications following aSAH of 160 patients with aSAH

	aSAH (*n* = 160)	DCI (*n* = 31)	No DCI (*n* = 129)	*p* value
Age, mean (SD), yr	57.8 (12.5)	58.0 (13.0)	57.8 (12.4)	0.92
Female, *n* (%)	114 (71)	27 (87)	87 (67)	0.05
Comorbidities, *n* (%)^a^				
Diabetes mellitus	5 (3)	2 (7)	3 (2)	0.24
Hypertension	48 (30)	13 (43)	35 (28)	0.12
Hypercholesterolemia	10 (6)	2 (7)	8 (6)	1.00
Cardiovascular disease	14 (9)	1 (3)	13 (10)	0.31
Smoking status, *n* (%)^b^				0.21
Current	56 (51)	13 (12)	43 (49)	
Previous	25 (23)	7 (6)	18 (21)	
Never	30 (27)	3 (3)	27 (31)	
Home medication, *n* (%)				
Antiplatelet	15 (9)	4 (3)	11 (7)	0.49
Anticoagulation	5 (3)	0 (0)	5 (4)	0.58
Tranexamic acid administration (ULTRA-trial intervention group), *n* (%)	26 (16)	4 (13)	22 (17)	0.78
WFNS, *n* (%)				0.76
Grade 1	50 (31)	8 (26)	42 (33)	
Grade 2	26 (16)	4 (13)	22 (17)	
Grade 3	5 (3)	1 (3)	4 (3)	
Grade 4	25 (16)	7 (23)	18 (14)	
Grade 5	54 (34)	11 (36)	43 (33)	
Fisher, *n* (%)				0.56
Grade 2	4 (3)	0 (0)	4 (3)	
Grade 3	31 (19)	7 (23)	24 (15)	
Grade 4	125 (78)	24 (77)	101 (78)	
Treatment modality, *n* (%)				0.03
Endovascular	124 (78)	29 (94)	95 (74)	
Stent-assisted coiling	3 (2)	1 (3)	2 (2)	
Flow diverter	2 (1)	1 (3)	1 (1)	
Surgical	14 (9)	2 (7)	12 (9)	
None	22 (14)	0 (0)	22 (17)	
Admission laboratory test result^c^				
Hemoglobin, mean (SD), mmol/L	8.3 (1.04)	8.1 (1.1)	8.3 (1.0)	0.52
Leukocytes, mean (SD), 10^9^/L	12.3 (5.1)	14.0 (6.4)	12.9 (4.6)	0.06
CRP, median (IQR), mg/L	2.0 (0.9–4.6)	2.6 (1.0–5.4)	1.9 (0.8–4.1)	0.25
Complications, *n* (%)				
Rebleeding	30 (19)	7 (23)	23 (18)	0.61
Hydrocephalus	113 (71)	25 (81)	88 (68)	0.20
CSF drainage	111	25	86	
Lumbar puncture(s)	25	11	14	
Treatment related				
Hemorrhagic complications	6 (4)	3 (10)	3 (3)	0.13
Thromboembolic complication	6 (4)	1 (3)	4 (4)	1.00
Cerebral ischemia	5 (4)	0 (0)	5 (6)	0.34
Meningitis	11 (7)	4 (13)	7 (5)	0.23
Pneumonia	21 (13)	3 (10)	18 (14)	0.77
Urinary tract infection	10 (6)	1 (1)	9 (7)	0.69
Seizures	22 (14)	4 (13)	18 (14)	1.00

aSAH, aneurysmal subarachnoid hemorrhage, CRP, C-reactive protein, DCI, delayed cerebral ischemia, IQR, interquartile range, WFNS, World Federation of Neurological Surgeons

^a^*n* = 3 (2%) missing

^b^*n* = 49 (31%) missing

^c^Hemoglobin: *n* = 14 (9%) missing; leukocytes: *n* = 13 (8%) missing; CRP: *n* = 55 (34%) missing

### DCI

EXTEM a-angle on admission was significantly higher in patients with clinical DCI than in patients without clinical DCI (*p* = 0.03), but all other parameters, including FIBTEM and INTEM parameters, did not differ between the groups at any time point (Supplemental Files, Fig. S1, page 10; Table S2A, page 3). PT (*p* = 0.04) and aPTT (*p* = 0.03) were significantly shorter in patients with clinical DCI, and D-dimer levels were significantly higher (*p* = 0.03) when compared to patients without DCI. After exclusion of patients who received tranexamic acid (*n* = 4 patients with DCI and *n* = 22 patients without DCI), the sensitivity analyses showed persistent hypercoagulable ROTEM profiles on admission and days 3–5 in patients with DCI compared to patients without DCI (Supplemental Files, Table S2B, page 4). In univariate logistic regression, none of the ROTEM parameters and conventional coagulation markers were significantly associated with a clinical diagnosis of DCI.

**Fig. 3:**
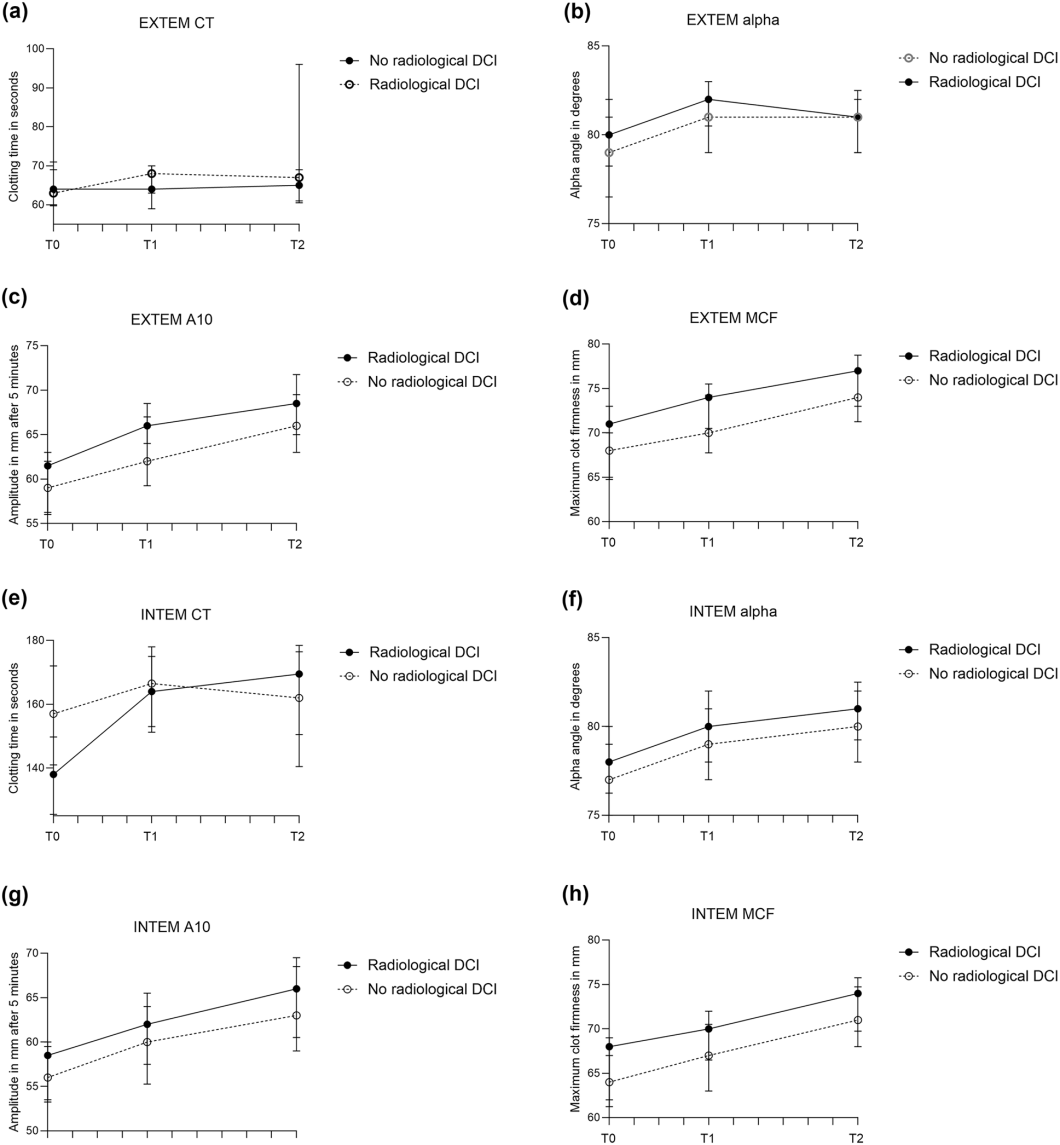

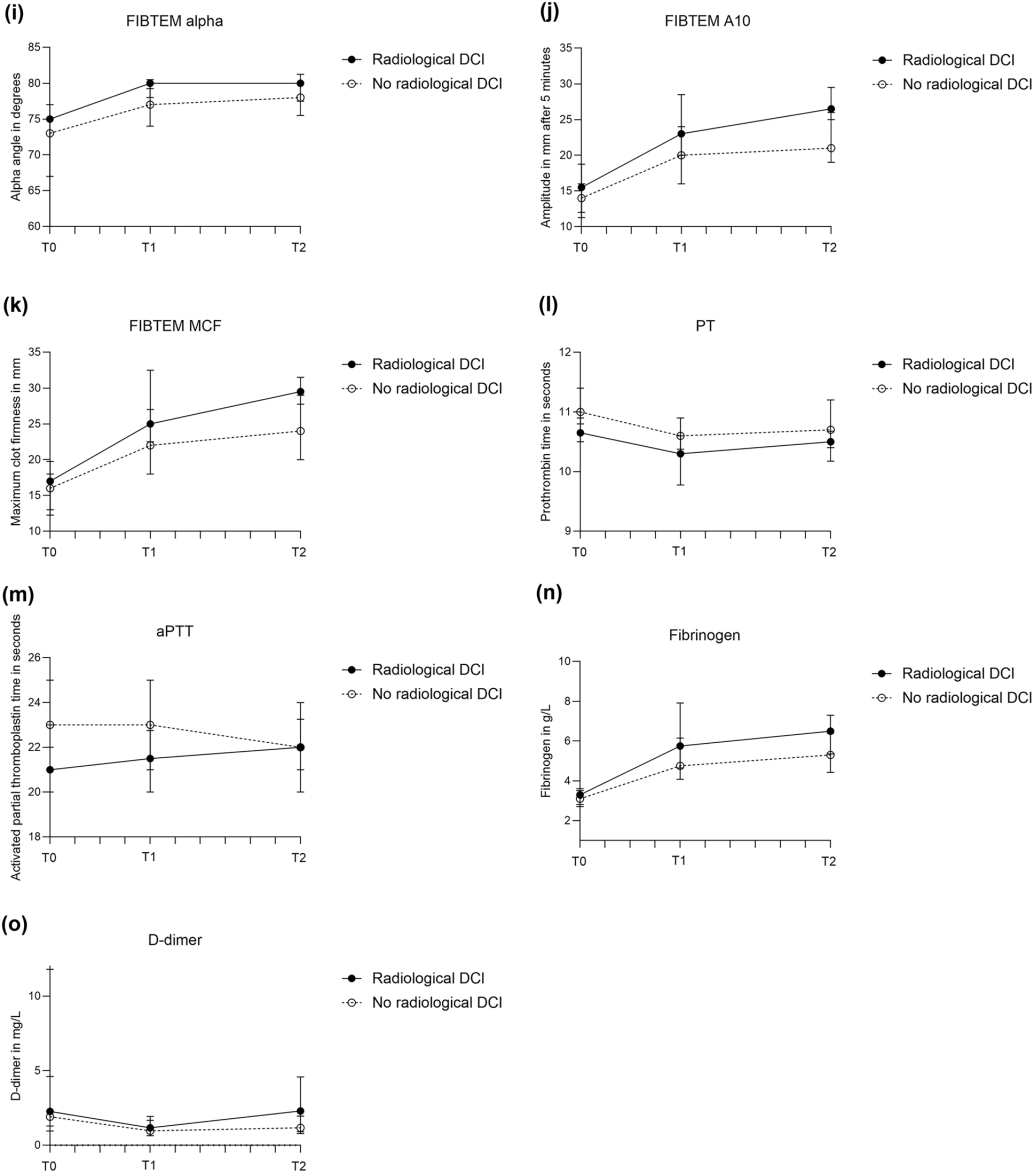
Medians (interquartile range) of rotational thromboelastometry (ROTEM) parameters in patients with and without radiological delayed cerebral ischemia (DCI), measured on admission, days 3–5, and days 9–11 after aneurysmal subarachnoid hemorrhage. **a,** EXTEM CT. **b,** EXTEM α-angle. **c,** EXTEM amplitude at 10 min (A10). **d,** EXTEM maximum clot firmness (MCF). **e,** INTEM CT. **f,** INTEM α-angle. **g,** INTEM A10. **h,** INTEM MCF. **i,** FIBTEM α-angle. **j,** FIBTEM A10. **k,** FIBTEM MCF. **l,** PT. **m,** aPTT. **n,** Fibrinogen. **o,** D-dimer. aPTT activated partial thromboplastin time, PT prothrombin time

Given the diagnostic challenges with DCI, also patients with radiological DCI were evaluated, and this comparison showed more clear differences between groups. INTEM CT on admission was significantly shorter in those with radiological DCI compared to those without radiological DCI (Fig. 3; medians [IQR] in Supplemental Files, Table S3A, page 5). On days 3–5, EXTEM CFT (*p* = 0.03), EXTEM a-angle (*p* = 0.04), EXTEM MCF (*p* = 0.05), FIBTEM a-angle (*p* = 0.02), FIBTEM A10 (*p* = 0.04), and FIBTEM MCF (*p* = 0.02) were all significantly different between groups, all suggesting more intense coagulation in patients developing radiological DCI. Of conventional coagulation markers, PT (*p* < 0.001) and aPTT (*p* = 0.007) on admission and PT (*p* = 0.02) and fibrinogen levels (*p* = 0.02) on days 3–5 were significantly different (Fig. 3; Supplemental Files, Table S3A, page 5). In the sensitivity analyses, in which patients who received tranexamic acid treatment were excluded (radiological DCI: n = 13, no radiological DCI: n = 121), patients with radiological DCI remained relatively hypercoagulable for most ROTEM parameters, but significance on days 3–5 was lost, likely due to a decrease in numbers (Supplemental Files, Table S3B, page 6). In univariate logistic regression, the ROTEM parameter with the highest AUC for radiological DCI was INTEM CT on admission (AUC 0.75, 95% CI 0.64–0.86), of which the optimal cutoff value was < 146 s (sensitivity 92%, specificity 20%, AUC values in Table 2; receiver operating characteristic [ROC] curves in Supplemental Files, Fig. S2, page 11).

**Table 2:** ORs and AUCs of ROTEM parameters on the occurrence of radiological DCI

Variable	OR (95% CI)	AUC (95% CI)
ROTEM parameters		
T0 INTEM CT	**0.96 (0.94–0.99)**	0.75 (0.64–0.86)
T1 EXTEM CFT	**0.94 (0.89–0.99)**	0.69 (0.56–0.82)
T1 EXTEM α-angle	1.43 (0.99–2.06)	–
T1 EXTEM MCF	1.16 (1.00–1.34)	–
T1 FIBTEM α-angle	1.25 (0.99–1.57**)**	–
T1 FIBTEM A10	**1.12 (1.01–1.24)**	0.71 (0.58–0.83)
T1 FIBTEM MCF	**1.12 (1.02–1.24)**	0.71 (0.58–0.84)
Conventional coagulation markers		
T0 PT	**0.15 (0.04–0.58)**	0.75 (0.64–0.86)
T0 aPTT	**0.68 (0.49–0.93)**	0.71 (0.57–0.84)
T1 PT	**0.18 (0.04–.0.77)**	0.70 (0.54–0.86)
T1 aPTT	**0.68 (0.47–0.99)**	0.70 (0.56–0.84)
T1 Fibrinogen	**1.55 (1.07–2.26)**	0.70 (0.55–0.86)

Bold valve indicate significant odds ratio, A10, amplitude at 10 min, aPTT activated partial thromboplastin time, AUC, area under the curve, CFT, clot formation time, CI, confidence interval, CT, clotting time, DCI, delayed cerebral ischemia, MCF, maximum clot firmness, OR, odds ratio, PT prothrombin time, ROTEM, rotational thromboelastometry, T0, day 0, T1, day 3–5

### Poor Clinical Outcome

**Fig. 4:**
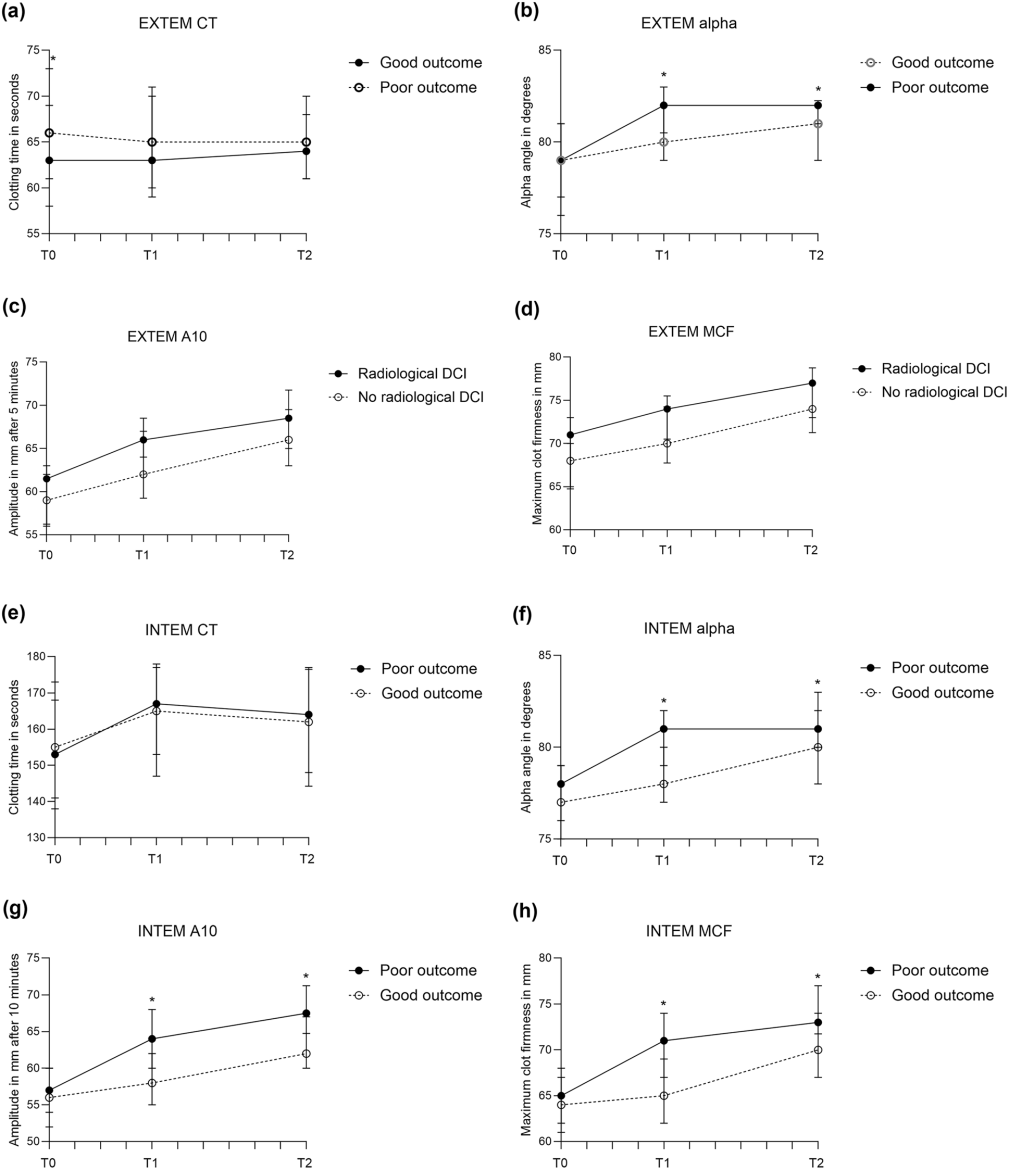

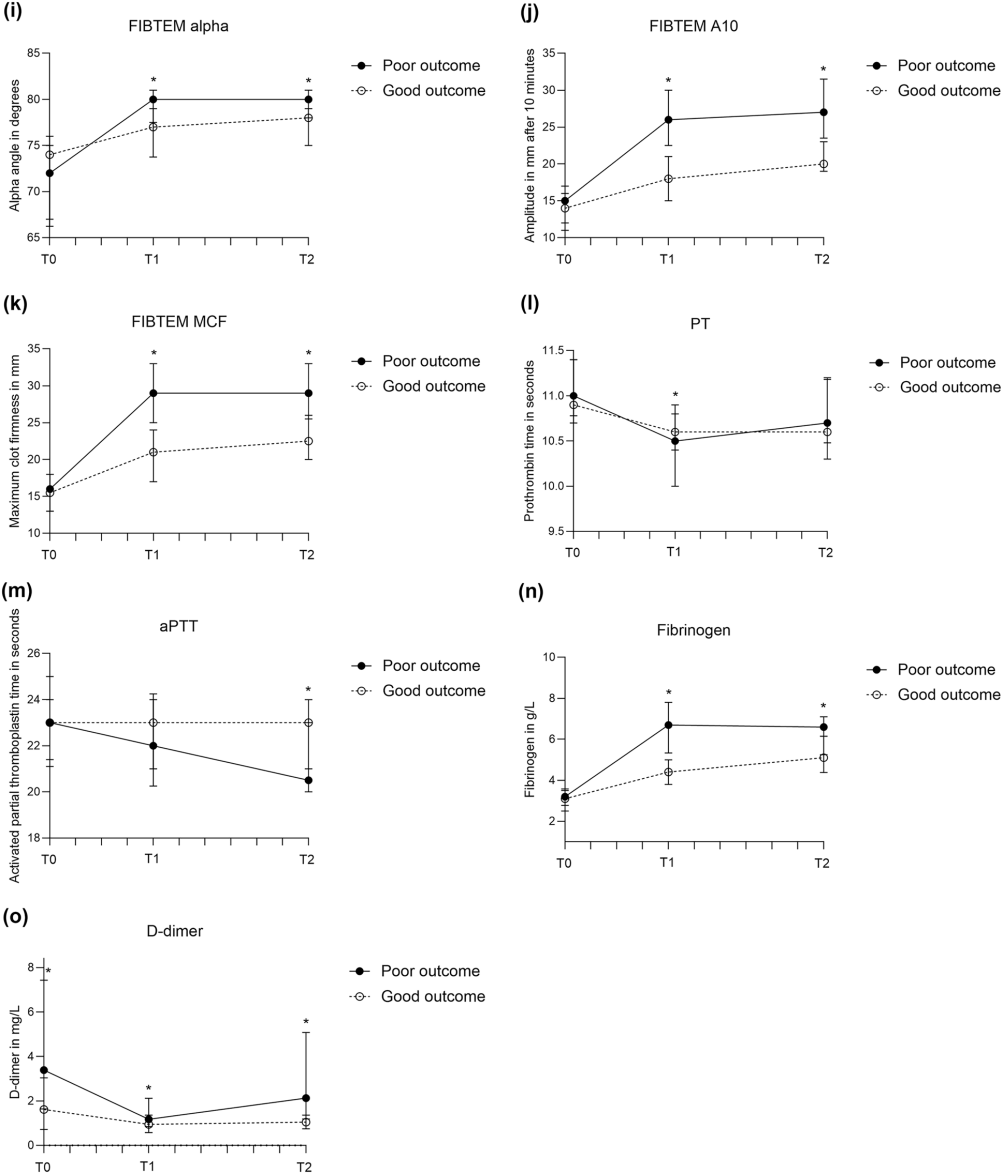
Medians (interquartile range) of rotational thromboelastometry (ROTEM) parameters in patients with good (mRS score 0–3) and poor (mRS score 4–6) clinical outcome at 6 months after aneurysmal subarachnoid hemorrhage (aSAH), measured on admission, days 3–5, and days 9–11 after aSAH. **a,** EXTEM CT. **b,** EXTEM α-angle. **c,** EXTEM amplitude at 10 min (A10). **d,** EXTEM maximum clot firmness (MCF). **e,** INTEM CT. **f,** INTEM α-angle. **g,** INTEM A10. **h,** INTEM MCF. **i,** FIBTEM α-angle. **j,** FIBTEM A10. **k,** FIBTEM MCF. **l,** PT. **m,** aPTT. **n,** Fibrinogen. **o,** D-dimer. aPTT activated partial thromboplastin time, mRS modified Rankin Scale, PT prothrombin time

The occurrence of common complications after aSAH in patients with good and poor clinical outcome are reported in the Supplemental Files, Table S4. Stent-assisted coiling and flow diverter placement were equally distributed among patients with good (two stent-assisted coiling, one flow diverter) and poor outcome (one stent-assisted coiling, one flow diverter). ROTEM profiles of patients with poor clinical outcome at 6 months were more hypercoagulable than in patients with good outcome*,* with increasing differences over time (Fig. 4, Table S3). Patients with poor outcome had significantly shorter EXTEM CT (*p* = 0.01) on admission when compared to patients with good outcome. On days 3–5 and 9–11, nearly all assessed ROTEM parameters showed more intense coagulation in patients with poor outcome compared to those with good outcome. Of conventional coagulation markers, D-dimer levels on admission (*p* < 0.001); PT (*p* = 0.03), fibrinogen levels (*p* ≤ 0.001), and D-dimer levels (*p* = 0.005) on days 3–5; and aPTT (*p* = 0.004), fibrinogen levels (*p* = 0.004), and D-dimer levels (*p* < 0.001) on days 9–11 were significantly different (Fig. 4; Table S5A). The sensitivity analyses, in which patients who received tranexamic acid treatment were excluded (poor outcome: *n* = 60, good outcome: *n* = 68), showed similar results with sustained statistical significance in patients with good and poor clinical outcome (Supplemental Files, Table S5B, page 7).

The results of the univariate logistic regression and ROC curve analyses are listed in Table 3 (ROC curves in Supplemental Files, Fig. S3, page 12). The ROTEM parameters with the highest AUC were FIBTEM A10 and FIBTEM MCF on days 3–5 (AUC of both parameters: 0.85, 95% CI 0.78–0.92; Table 3). The optimal cutoff value of FIBTEM A10 was > 27 mm (specificity 94%, sensitivity 49%). The optimal cutoff value of FIBTEM MCF was > 30 mm (specificity 91%, sensitivity 46%).

**Table 3:** ORs and AUCs of ROTEM parameters on poor clinical outcome

Variable	OR (95% CI)	AUC (95% CI)
ROTEM parameters		
T0 EXTEM CT	1.02 (0.99–1.04)	**–**
T1 EXTEM CFT	**0.91 (0.87–0.95)**	0.79(0.71–0.88)
T1 EXTEM α-angle	**1.72 (1.32–2.24)**	0.76 (0.66–0.85)
T1 EXTEM A10	**1.27 (1.15–1.41)**	0.79 (0.71–0.88)
T1 EXTEM MCF	**1.36 (1.20–1.54)**	0.80 (0.72–0.88)
T1 INTEM CFT	**0.94 (0.91–0.97)**	0.76 (0.67–0.86)
T1 INTEM α-angle	**1.47 (1.23–1.77)**	0.76 (0.66–0.85)
T1 INTEM A10	**1.21 (1.12–1.32)**	0.77 (0.68–0.86)
T1 INTEM MCF	**1.30 (1.16–1.45)**	0.79 (0.71–0.88)
T1 FIBTEM α-angle	**1.37 (1.17–1.61)**	0.76 (0.67–0.85)
T1 FIBTEM A10	**1.31 (1.18–1.45)**	0.85 (0.78–0.92)
T1 FIBTEM MCF	**1.28 (1.17–1.40)**	0.85 (0.78–0.92)
T2 EXTEM CFT	**0.93 (0.86–0.99)**	0.74 (0.60–0.87)
T2 EXTEM α-angle	**1.46 (1.02–2.09)**	0.71 (0.57–0.85)
T2 EXTEM A10	**1.18 (1.03–1.34)**	0.74 (0.61–0.88)
T2 EXTEM MCF	**1.23 (1.04–1.45)**	0.71 (0.57–0.85)
T2 INTEM CFT	**0.95 (0.91–0.998)**	0.68 (0.54–0.83)
T2 INTEM α-angle	**1.35 (1.04–1.77)**	0.68 (0.54–0.82)
T2 INTEM A10	**1.14 (1.02–1.28)**	0.72 (0.58–0.87)
T2 INTEM MCF	**1.19 (1.03–1.38)**	0.71 (0.57–0.85)
T2 FIBTEM α-angle	**1.39 (1.08–1.79)**	0.72 (0.59–0.86)
T2 FIBTEM A10	**1.27 (1.11–1.46)**	0.82 (0.72–0.93)
T2 FIBTEM MCF	**1.26 (1.10–1.43)**	0.82 (0.72–0.93)
Conventional coagulation markers		
T0 D-dimer	**1.11 (1.04–1.18)**	0.69 (0.60–0.78)
T1 PT	**0.45 (0.21–0.94)**	0.63 (0.52–0.73)
T1 Fibrinogen	**2.43 (1.74–3.40)**	0.84 (0.77–0.92)
T1 D-dimer	1.26 (0.92–1.74)	–
T2 aPTT	0.62 (0.43–0.90)	–
T2 Fibrinogen	**1.87 (1.18–2.96)**	0.73 (0.60–0.86)
T2 D-dimer	1.02 (0.99–1.04)	–

Bold valve indicate significant odds ratio, A10, amplitude at 10 min, aPTT activated partial thromboplastin time, AUC, area under the curve, CFT, clot formation time, CI, confidence interval, CT, clotting time, MCF, maximum clot firmness, OR, odds ratio, PT prothrombin time, ROTEM, rotational thromboelastometry, T0, day 0, T1, day 3-5, T2, day 9–11

## Discussion

Throughout the entire time course after aSAH, ROTEM profiles of patients with DCI or poor outcome were more coagulable when compared to those of patients without these complications. For radiological DCI, short INTEM CT already at hospital admission and high FIBTEM a-angle, A10, and MCF on days 3–5 had an acceptable discriminative ability (AUC > 0.70). For poor outcome, nearly all markers of increased clot kinetics and clot strength had an AUC > 0.70.

We examined prognostic utility of ROTEM for DCI because enhanced coagulation contributes to the development of DCI, and viscoelastic testing is currently a valuable test used in clinical practice that has the ability to detect hypercoagulability. Indeed, we show that patients who developed DCI had intensified ROTEM coagulation profiles then patients who did not develop DCI. Three studies have been published on ROTEM and DCI. Additionally, three studies on thromboelastography, a viscoelastic test comparable to ROTEM, were conducted on patients with aSAH and DCI. Four of six studies showed that ROTEM parameters, especially markers of clot strength, are significantly different in patients with DCI compared to those without DCI, with relatively hypercoagulable ROTEM profiles in patients with DCI [13, 21]. Differences between groups were more pronounced when radiological DCI as an outcome was evaluated instead of a clinical diagnosis of DCI. This may be explained by the use of objective diagnostic criteria, which likely reduced heterogeneity of patients with clinical DCI, who may actually have had complications other than cerebral ischemia. Conventional markers such as PT, aPTT, and fibrinogen measured early after ictus also indicated enhanced coagulation in those developing DCI, but these differences disappeared later in the course of SAH, whereas differences in ROTEM parameters became more outspoken. In line with these findings, previous studies on associations between DCI and PT or aPTT have shown inconsistent results [11].

Differences in ROTEM profiles became even more pronounced between patient with good and poor 6-month outcome. In patients with poor outcome, almost all ROTEM parameters pointed toward hypercoagulability, including parameters of clot kinetics and clot strength, which suggests increased platelet activity as well as fibrinogen contribution to clot formation. This is in line with previous literature, which has consequently shown a significant hypercoagulable profile in patients with poor clinical outcome compared to those with good clinical outcome [13, 21]. Our study is the first to investigate the predictive ability of ROTEM for clinical outcome. Of interest is that FIBTEM markers yielded the highest AUCs, with comparable discriminative ability to the WFNS grade, which is the current most commonly used prognosticator [22, 23]. The contribution of fibrinogen to poor clinical outcome in aSAH may also be in line with recent insights into the role of fibrinogen in amplifying immune and degenerative processes in the brain in various neurological disorders [24].

Whether correction of the observed relative hypercoagulability will result in a lower proportion of patients with DCI or improved clinical outcome is yet unknown. Based on our results, it is tempting to speculate that antiplatelet drugs, fibrinogen-depleting drugs, or inhibitors of fibrin formation might be potential treatment strategies to reduce poor clinical outcome after aSAH. In the past, a Cochrane review on antiplatelet therapy after SAH showed a nonsignificant trend toward a lower occurrence of DCI and improved clinical outcome but was possibly offset by a concomitant nonsignificant increase in hemorrhagic complications [25]. ROTEM might therefore be helpful in stratifying patients with hypercoagulability for therapeutic interventions, without exposing patients without hypercoagulability to an increased risk of hemorrhagic complications.

Another area in which results could be useful is for monitoring. In current clinical practice, all patients with SAH are hospitalized for a minimum of 2 weeks to monitor complications, all of which drive poor outcome. We found that ROTEM has a high discriminative ability to predict 6-month poor outcome, using a sensitivity threshold of at least 90%, which is a high likelihood of the absence of complications. However, to discharge patients at low risk of DCI or other complications or to guide physicians to determine the futility of care, an even higher sensitivity would be preferable.

In studies such as these, it is difficult to correct for confounders. An obvious confounder in our study was tranexamic acid because a study on this drug was concurrently ongoing. However, in a sensitivity analysis in which patients who received tranexamic acid were excluded, the ability of ROTEM to detect poor outcome was maintained, whereas its ability to detect DCI was lost. The latter may be explained by a loss of power resulting from smaller sample sizes. Potential confounding by tranexamic acid is less likely, as the proportion of patients treated with tranexamic acid in patients with and without DCI was similar, and patients with DCI remained relatively hypercoagulable in comparison to patients without DCI. Also, patients treated with and without tranexamic acid had similar ROTEM coagulation profiles, with only three parameters on admission with significant differences. Twenty-nine of 30 ROTEM parameters with significant differences either for radiological DCI or for poor outcome were measured on days 3–5 and 9–11. Other confounders were not accounted for, such as WFNS grade, age, treatment modality, or other SAH-related complications that may influence clinical outcome. Also, we did not correct for multiple testing, given that parameters are interrelated. Thereby, the risk of a chance finding is present.

Another consideration is that the INTEM CT cutoff value (< 146 s) we found is within the reference range. Thereby, one may question whether patients with DCI can be referred to as “hypercoagulable.” However, also in patients with sepsis, ROTEM can detect diffuse intravascular coagulation, which is an obvious pathological condition, while partly being between reference values [26]. This raises the question of whether reference values as derived from relatively small numbers of healthy individuals are helpful to set the boundaries of “normal.” In our opinion, relating ROTEM values to abnormalities in specific disease conditions may be more helpful.

These uncertainties taken together, validation studies are needed. Strengths of our study are the prospective nature, the use of standardized outcome measurements, and longitudinally measured ROTEM parameters following aSAH. To the best of our knowledge, this is the first study to assess the discriminative ability of ROTEM parameters for DCI and poor clinical outcome with clear cutoff values.

## Conclusions

In this study, ROTEM parameters suggesting enhanced coagulation have good predictive ability for poor clinical outcome. If independently validated, ROTEM parameters might have the potential to stratify patients with aSAH who may benefit from anticoagulant treatment in future prospective clinical trials. Additionally, there may be potential for ROTEM parameters to aid in earlier discharge decision-making that impacts hospital length of stay and resource use. Given the small sample size and presence of multiple confounders, our findings are purely hypothesis generating, and validation studies using larger prospective, ideally randomized controlled aSAH cohorts are needed.

## Conflict of interest

NPJ reported that the institution has received honoraria from Octapharma for unrestricted investigator-initiated research and lectures and that she is an editor-in-chief of *Intensive Care Medicine*. NPJ and WPV reported to have received a PoC grant of Amsterdam Neuroscience for this study. All other authors have disclosed that they do not have any conflicts of interest.

## Ethical Approval/Informed Consent

The study was conducted in accordance with the Declaration of Helsinki. The protocol was approved by our institutional review board (MEC 2017_318). Deferred informed consent was obtained from patients or their legal representatives, except those who suffered imminent death. Standard of care consisted of securing the aneurysm < 24–72 h by endovascular coiling or surgical clipping.

## Consent for Publication

A previous version of the manuscript has been uploaded by preprint services. However, this manuscript has not been published elsewhere and is not under consideration by another journal

## Supplementary Information

Below is the link to the electronic supplementary material.

Supplementary file1 (DOCX 729 KB)

## Data Availability

Access to anonymized data may be granted following review. All data requests should be submitted to the first author (MAT).

## References

[1] Aneurysmal DS, Hemorrhage S. Aneurysmal Subarachnoid Hemorrhage. J Neurosurg Anesthesiol. 2015;27(3):222–40. 25272066 10.1097/ANA.0000000000000130 PMC4463029

[2] Stienen MN, Germans M, Burkhardt J-K, Neidert MC, Fung C, Bervini D, et al. Predictors of in-hospital death after aneurysmal subarachnoid hemorrhage. Stroke. 2018;49(2):333–40. 29335333 10.1161/STROKEAHA.117.019328

[3] Kassell NF, Torner JC, Haley EC Jr, Jane JA, Adams HP, Kongable GL. The international cooperative study on the timing of aneurysm surgery. Part 1: Overall management results. J Neurosurg. 1990;73(1):18–36. 2191090 10.3171/jns.1990.73.1.0018

[4] Hop JW, Rinkel GJ, Algra A, van Gijn J. Case-fatality rates and functional outcome after subarachnoid hemorrhage: a systematic review. Stroke. 1997;28(3):660–4. 9056628 10.1161/01.str.28.3.660

[5] Roos Y, De Haan R, Beenen L, Groen R, Albrecht K, Vermeulen M. Complications and outcome in patients with aneurysmal subarachnoid haemorrhage: a prospective hospital based cohort study in the Netherlands. J Neurol Neurosurg Psychiatr. 2000;68(3):337–41. 10.1136/jnnp.68.3.337 PMC1736841 10675216

[6] Budohoski KP, Guilfoyle M, Helmy A, Huuskonen T, Czosnyka M, Kirollos R, et al. The pathophysiology and treatment of delayed cerebral ischaemia following subarachnoid haemorrhage. J Neurol Neurosurg Psychiatr 2014;307711. 10.1136/jnnp-2014-307711 24847164

[7] Vergouwen MD, Vermeulen M, Coert BA, Stroes ES, Roos YB. Microthrombosis after aneurysmal subarachnoid hemorrhage: an additional explanation for delayed cerebral ischemia. J Cereb Blood Flow Metab. 2008;28(11):1761–70. 18628782 10.1038/jcbfm.2008.74

[8] Hoh BL, Ko NU, Amin-Hanjani S, Chou SHY, Cruz-Flores S, Dangayach NS, et al. Guideline for the management of patients with aneurysmal subarachnoid hemorrhage: a guideline from the American heart association/American stroke association. Stroke 2023. 10.1161/STR.0000000000000436 37212182

[9] Treggiari MM, Rabinstein AA, Busl KM, Caylor MM, Citerio G, Deem S, et al. Guidelines for the neurocritical care management of aneurysmal subarachnoid hemorrhage. Neurocrit Care 2023. 10.1007/s12028-023-01713-5 37202712

[10] Steiner T, Juvela S, Unterberg A, Jung C, Forsting M, Rinkel G. European stroke organization guidelines for the management of intracranial aneurysms and subarachnoid haemorrhage. Cerebrovasc Dis. 2013;35(2):93–112. 23406828 10.1159/000346087

[11] Boluijt J, Meijers JC, Rinkel GJ, Vergouwen MD. Hemostasis and fibrinolysis in delayed cerebral ischemia after aneurysmal subarachnoid hemorrhage: a systematic review. J Cereb Blood Flow Metab. 2015;35(5):724–33. 25690473 10.1038/jcbfm.2015.13 PMC4420861

[12] Luddington RJ. Thrombelastography/thromboelastometry. Clin Lab Haematol. 2005;27(2):81–90. 15784122 10.1111/j.1365-2257.2005.00681.x

[13] Tjerkstra MA, Wolfs AE, Verbaan D, Vandertop WP, Horn J, Müller MCA, Juffermans NP. A systematic review on viscoelastic testing in subarachnoid haemorrhage patients. World Neurosurg 2023. 10.1016/j.wneu.2023.03.108 37004882

[14] Post R, Germans MR, Tjerkstra MA, Vergouwen MDI, Jellema K, Koot RW, et al. Ultra-early tranexamic acid after subarachnoid haemorrhage (ULTRA): a randomised controlled trial. The Lancet. 2021;397(10269):112–8. 10.1016/S0140-6736(20)32518-6 33357465

[15] Vergouwen MD, Vermeulen M, van Gijn J, Rinkel GJ, Wijdicks EF, Muizelaar JP, et al. Definition of delayed cerebral ischemia after aneurysmal subarachnoid hemorrhage as an outcome event in clinical trials and observational studies: proposal of a multidisciplinary research group. Stroke. 2010;41(10):2391–5. 20798370 10.1161/STROKEAHA.110.589275

[16] Baksaas-Aasen K, Van Dieren S, Balvers K, Juffermans NP, Næss PA, Rourke C, et al. Data-driven Development of ROTEM and TEG algorithms for the management of trauma hemorrhage: a prospective observational multicenter study. Ann Surg. 2019;270(6):1178–85. 29794847 10.1097/SLA.0000000000002825

[17] Hosmer Jr DW, Lemeshow S, Sturdivant RX. Applied logistic regression. Wiley; 2013.

[18] Deeks JJ, Altman DG. Diagnostic tests 4: likelihood ratios. BMJ. 2004;329(7458):168–9. 15258077 10.1136/bmj.329.7458.168 PMC478236

[19] Ranganathan P, Aggarwal R. Common pitfalls in statistical analysis: understanding the properties of diagnostic tests—Part 1. Perspect Clin Res. 2018;9(1):40–3. 29430417 10.4103/picr.PICR_170_17 PMC5799952

[20] Ranganathan P, Aggarwal R. Understanding the properties of diagnostic tests - Part 2: likelihood ratios. Perspect Clin Res. 2018;9(2):99–102. 29862204 10.4103/picr.PICR_41_18 PMC5950618

[21] Raatikainen E, Kiiski H, Kuitunen A, Junttila E, Huhtala H, Kallonen A, et al. Increased blood coagulation is associated with poor neurological outcome in aneurysmal subarachnoid hemorrhage. J Neurol Sci. 2024;458: 122943. 38422781 10.1016/j.jns.2024.122943

[22] Nguyen TA, Vu LD, Mai TD, Dao CX, Ngo HM, Hoang HB, et al. Predictive validity of the prognosis on admission aneurysmal subarachnoid haemorrhage scale for the outcome of patients with aneurysmal subarachnoid haemorrhage. Sci Rep. 2023;13(1):6721. 37185953 10.1038/s41598-023-33798-5 PMC10130082

[23] van Heuven AW, Dorhout Mees SM, Algra A, Rinkel GJ. Validation of a prognostic subarachnoid hemorrhage grading scale derived directly from the Glasgow Coma Scale. Stroke. 2008;39(4):1347–8. 18309166 10.1161/STROKEAHA.107.498345

[24] Petersen MA, Ryu JK, Akassoglou K. Fibrinogen in neurological diseases: mechanisms, imaging and therapeutics. Nat Rev Neurosci. 2018;19(5):283–301. 29618808 10.1038/nrn.2018.13 PMC6743980

[25] Dorhout Mees SM, Van Den Bergh WM, Algra A, Rinkel GJE. Antiplatelet therapy for aneurysmal subarachnoid haemorrhage. Cochrane Database of Systematic Reviews 2007;(2) (no pagination)(CD006184). 10.1002/14651858.CD006184.pub2 PMC8919458 17943892

[26] Muller MC, Meijers JC, Vroom MB, Juffermans NP. Utility of thromboelastography and/or thromboelastometry in adults with sepsis: a systematic review. Crit Care. 2014;18(1):R30. 24512650 10.1186/cc13721 PMC4056353

