# Supplementary material for: The Discriminative Ability of ROTEM for Delayed Cerebral Ischemia and Poor Outcome Following Aneurysmal Subarachnoid Hemorrhage
Source: Neurocrit Care. 2025 Jul 16;43(3):999–1012. doi: 10.1007/s12028-025-02309-x (PMC12647204; doi:10.1007/s12028-025-02309-x)
Supplement: Supplementary file 1 — Supplementary file1 (DOCX 729 KB) [file 12028_2025_2309_MOESM1_ESM.docx]

# Supplemental Files

## Table of contents

Methods: definitions of complications Page 1

Table S1: Trajectory of ROTEM-parameters in aSAH patients treated with and without Page 2
tranexamic acid

Table S2A: Trajectory of ROTEM-parameters in patients with and without DCI Page 3

Table S2B: Trajectory of ROTEM-parameters in patients with and without DCI, Page 4
after exclusion of patients treated with tranexamic acid

Table S3A: Trajectory of ROTEM-parameters in patients with and without Page 5
radiological DCI

Table S3B: Trajectory of ROTEM-parameters in patients with and without Page 6
radiological DCI, after exclusion of patients treated with tranexamic acid

Table S4 Frequency of common complications of aSAH in patients with good and Page 7
poor clinical outome

Table S5A: Trajectory of ROTEM-parameters in patients with good and poor clinical Page 8
outcome at six months post-SAH

Table S5B: Trajectory of ROTEM-parameters in patients with good and poor clinical Page 9
outcome at six months post-SAH, after exclusion of patients treated with tranexamic acid

Figure S1: Medians (IQR) of ROTEM-parameters in patients with and without Page 10
DCI, measured on admission, days 3-5 and days 9-11 after aSAH.

Figure S2: ROC-curves on radiological DCI of ROTEM-parameters with a significant Page 11
association with radiological DCI in univariate logistic regression analyses.

Figure S3: ROC-curves on poor clinical outcome of ROTEM-parameters with a Page 12-13
a significant association with poor clinical outcome in univariate logistic regression
analyses.

**Definitions of complications**

Rebleeding was scored when patients suddenly deteriorated in the presence of an increase of blood on CT-imaging compared to a previous investigation.

Hemorrhagic complications were defined as either extravasation of contrast dye or perforation during endovascular treatment or excessive bleeding during neurosurgical treatment.

Thrombo-embolic complications were defined as reduced passage or stasis of contrast in an artery or slowed venous outflow without the aspect of vascular spasm.

Ischemic complications were defined as post-procedural neurological deterioration, in combination with new ischemic lesions on post-operative imaging.

Hydrocephalus was defined as deterioration of consciousness with either CT evidence of either enlarged ventricles or increased intracranial pressure measured by a lumbar puncture.

Seizures were scored based on clinical evaluation.

Meningitis was defined as a positive cerebrospinal fluid culture.

Pneumonia was defined as either a positive sputum culture and/or an infiltrate on chest x-ray.

Urinary tract infection was defined as a positive urine culture or abnormal urine sample.

**Table S1: Trajectory of ROTEM-parameters of patients treated with and without tranexamic acid.**

|  | **T0** n=157 |  |  | **T1** n=125 |  |  | **T2** n=63 |  |  |
| --- | --- | --- | --- | --- | --- | --- | --- | --- | --- |
|  | **TXA** (n=25) | **No TXA** (n=132) | **p-value** | **TXA** (n=25) | **No TXA** (n=100) | **p-value** | **TXA** (n=21) | **No TXA** (n=42) | **p-value** |
| **EXTEM CT** | 61 (58-67) | 64 (60-71) | 0.13^†^ | 67 (59-74) | 64 (59-70) | 0.35 | 62 (58-65) | 67 (62-71) | 0.14^†^ |
| **EXTEM CFT** | 59 (51-68) | 62 (54-75) | 0.28 | 80 (79-82) | 81 (79-82) | 0.86^†^ | 47 (40-52) | 46 (9-57) | 0.54^†^ |
| **EXTEM α-angle** | 80 (77-81) | 79 (77-81) | 0.52 | 63 (61-67) | 63 (60-67) | 0.50^†^ | 81 (80-82) | 81 (79-82) | 0.71^†^ |
| **EXTEM A10** | 60 (58-62) | 59 (55-62) | 0.12 | 55 (40-64) | 50 (42-63) | 0.67^†^ | 66 (63-70) | 67 (63-70) | 0.93^†^ |
| **EXTEM MCF** | 69 (67-72) | 68 (65-70) | 0.12 | 72 (68-75) | 70 (68-75) | 0.87^†^ | 75 (72-76) | 75 (71-78) | 0.75 |
| **EXTEM LI60** | 99 (97-99) | 99 (97-99) | 0.83 | 97 (94-99) | 97 (95-98) | 0.66^†^ | 98 (97-99) | 98 (97-99) | 0.66 |
|  |  |  |  |  |  |  |  |  |  |
| **INTEM CT** | 145 (134-166) | 155 (141-172) | 0.12^†^ | 161 (153-179) | 167 (151-178) | 0.79^†^ | 162 (146-173) | 164 (154-178) | 0.37^†^ |
| **INTEM CFT** | 63 (57-70) | 68 (57-81) | 0.08 | 62 (43-76) | 60 (46-73) | 0.84^†^ | 50 (41-57) | 48 (39-65) | 0.58^†^ |
| **INTEM α-angle** | **78 (77-81)** | **77 (75-79)** | **0.02** | 78 (77-82) | 79 (77-81) | 0.81^†^ | 80 (79-82) | 80,5 (78-82) | 0.68^†^ |
| **INTEM A10** | 57 (55-60) | 56 (53-60) | 0.12 | 60 (55-65) | 60 (56-65) | 0.95^†^ | 63 (62-69) | 63,5 (60-68) | 0.76^†^ |
| **INTEM MCF** | 66 (64-68) | 64 (61-68) | 0.12^†^ | 68 (65-70) | 67 (63-71) | 0.97^†^ | 72 (68-74) | 71 (67-75) | 0.88 |
| **INTEM LI60** | 98 (96-99) | 98 (96-99) | 0.84 | 97 (93-99) | 97 (95-98) | 0.84^†^ | 97 (95-99) | 98 (96-99) | 0.96^†^ |
|  |  |  |  |  |  |  |  |  |  |
| **FIBTEM α-angle** | 74 (70-77) | 72 (66-76) | 0.56 | 76 (75-81) | 78 (75-80) | 0.56 | 79 (75-81) | 78 (76-80) | 0.79^†^ |
| **FIBTEM A10** | **15 (13-18)** | **14 (11-16)** | **0.03^†^** | 20 (17-27) | 20 (16-26) | 0.95^†^ | 21 (19-28) | 22 (20-26) | 0.73 |
| **FIBTEM MCF** | **17 (13-21)** | **16 (13-18)** | **0.04^†^** | 24 (18-30) | 23 (19-28) | 0.85^†^ | 24 (21-29) | 25 (21-29) | 0.96^†^ |
|  |  |  |  |  |  |  |  |  |  |
| **PT** | 11.0 (10.7-11.3) | 10.9 (10.7-11.4) | 0.90 | 10.8 (10.3-11.5) | 10.6 (10.3-10.9) | 0.10 | 10.5 (10.4-11.2) | 10.7 (10.4-11.2) | 0.49^†^ |
| **aPTT** | 23 (22-24) | 23 (21-25) | 0.56 | 23 (21-245) | 22 (21-24) | 0.90 | 22 (20-24) | 22 (21-24) | 0.28^†^ |
| **Fibrinogen** | **3.5 (2.9-4.0)** | **3.1 (2.7-3.5)** | **0.03**^†^ | 5.0 (4.2-6.9) | 4.8 (4.1-6.4) | 0.67^†^ | 5.4 (4.5-6.3) | 5.6 (4.5-6.9) | 0.57^†^ |
| **D-dimer** | **1.4 (0.6-2.8)** | **2.3 (1.1-5.4)** | **0.03**^†^ | **0.8 (0.5-1.2)** | **1.1 (0.7-1.8)** | **0.04** | 1.4 (0.8-1.9) | 1.2 (0.8-2.3) | 0.65 |

All variables are displayed as median (IQR) regardless of the distribution of the data. Variables with normal distribution are, however, statistically tested using the T-test (indicated by †)
Data unavailable of 1 to 5 patients for MCF measurements, 1 to 6 patients for FIBTEM & α-angle measurements, 3 to 6 patients for LI60 measurements and 1 to 7 patients for PT, APTT, fibrinogen or D-dimer.

Reference values

|  | CT (s) | CFT (s) | α (hoek) | A10 (mm) | MCF (mm) |
| --- | --- | --- | --- | --- | --- |
| INTEM | 100-240 | 30-110 | 70-83 | 48-69 | 50-72 |
| EXTEM | 38-79 | 34-159 | 63-83 | 48-69 | 50-72 |
| IBTEM | - | - | - | 7-23 | - |

**Table S2A: Trajectory of ROTEM-parameters in patients with and without DCI.**

|  | **T0** n=157 |  |  | **T1** n=125 |  |  | **T2** n=63 |  |  |
| --- | --- | --- | --- | --- | --- | --- | --- | --- | --- |
|  | **DCI +** (n=31) | **DCI -** (n=129) | **p-value** | **DCI +** (n=26) | **DCI -** (n=99) | **p-value** | **DCI +** (n=12) | **DCI -** (n=51) | **p-value** |
| **EXTEM CT** | 63 (62-71) | 64 (60-70) | 0.12^†^ | 68 (62-71) | 63 (59-70) | 0.15 | 67 (63-71) | 64 (59-69) | 0.36^†^ |
| **EXTEM CFT** | 55 (49-72) | 63 (54-74) | 0.08 | 46 (39-54) | 52 (42-65) | 0.06^†^ | 42 (36-57) | 47 (40-54) | 0.52^†^ |
| **EXTEM α-angle** | **80 (78-82)** | **79 (76-80)** | **0.03** | 82 (80-82) | 81 (79-82) | 0.15^†^ | 82 (79-83) | 81 (79-82) | 0.77^†^ |
| **EXTEM A10** | 61 (56-63) | 59 (56-62) | 0.24 | 65 (62-67) | 62 (59-67) | 0.11^†^ | 69 (63-74) | 66 (63-69) | 0.38^†^ |
| **EXTEM MCF** | 69 (64-72) | 68 (65-70) | 0.13 | 72 (70-75) | 70 (68-75) | 0.07^†^ | 77 (72-80) | 74 (71-77) | 0.18 |
| **EXTEM LI60** | 99 (97-99) | 99 (97-99) | 0.47 | 97 (95-98) | 97 (95-98) | 0.38^†^ | 99 (97-99) | 98 (97-99) | 0.22 |
|  |  |  |  |  |  |  |  |  |  |
| **INTEM CT** | 149 (136-164) | 157 (141-172) | 0.30^†^ | 164 (156-173) | 167 (149-179) | 0.84^†^ | 160 (150-178) | 165 (148-177) | 0.66^†^ |
| **INTEM CFT** | 64 (52-79) | 68 (58-80) | 0.24 | 51 (43-69) | 62 (47-75) | 0.24^†^ | 45 (35-63) | 50 (41-60) | 0.56^†^ |
| **INTEM α-angle** | 78 (75-80) | 77 (75-79) | 0.31 | 80 (78-82) | 79 (77-81) | 0.13^†^ | 81 (78-83) | 80 (78-82) | 0.38^†^ |
| **INTEM A10** | 57 (53-60) | 56 (54-59) | 0.44 | 62 (56-65) | 60 (56-64) | 0.47^†^ | 66 (61-71) | 63 (61-68) | 0.76^†^ |
| **INTEM MCF** | 66 (62-69) | 64 (61-67) | 0.19^†^ | 68 (65-71) | 67 (63-71) | 0.43^†^ | 74 (69-78) | 71 (68-74) | 0.20 |
| **INTEM LI60** | 99 (96-100) | 98 (96-99) | 0.28 | 97 (94-98) | 97 (95-99) | 0.80^†^ | 99 (97-100) | 98 (96-99) | 0.34^†^ |
|  |  |  |  |  |  |  |  |  |  |
| **FIBTEM α-angle** | 73 (64-76) | 73 (67-75) | 0.85 | 79 (76-80) | 77 (74-79) | 0.05 | 80 (77-81) | 78 (76-80) | 0.48^†^ |
| **FIBTEM A10** | 15 (11-17) | 14 (12-16) | 0.66^†^ | 22 (20-27) | 20 (16-25) | 0.14^†^ | 26 (21-28) | 21 (19-26) | 0.26 |
| **FIBTEM MCF** | 17 (13-19) | 16 (13-18) | 0.44^†^ | 25 (22-30) | 23 (18-27) | 0.12^†^ | 29 (23-31) | 24 (21-29) | 0.26^†^ |
|  |  |  |  |  |  |  |  |  |  |
| **PT** | **10.8 (10.6-11.2)** | **11.0 (10.8-11.4)** | **0.04** | 10.6 (10.1-10.8) | 10.6 (10.4-10.9) | 0.22 | 10.5 (10.3-10.8) | 10.7 (10.4-11.2) | 0.10^†^ |
| **aPTT** | **22 (21-24)** | **23 (22-25)** | **0.03** | 22 (20-24) | 23 (21-25) | 0.07 | 22 (20-24) | 22 (21-24) | 0.40^†^ |
| **Fibrinogen** | 3,3 (2,8-3,7) | 3.1 (2.7-3.5) | 0.24^†^ | 5.3 (4.3-6.7) | 4.8 (4.0-6.4) | 0.24^†^ | 5.5 (4.8-7.0 | 5.5 (4.5-6.5) | 0.87^†^ |
| **D-dimer** | 2,1 (1,2-6.0) | 2.0 (0.95-4.6) | 0.49^†^ | 1.2 (0.8-2.1) | 1.0 (0.7-1.6) | 0.11 | **1.7 (1.0-4.2)** | **1.1 (0.7-1.9)** | **0.03** |

All variables are displayed as median (IQR) regardless of the distribution of the data. Variables with normal distribution are, however, statistically tested using the T-test (indicated by †)
Data unavailable of 1 to 5 patients for MCF measurements, 2 to 5 patients for FIBTEM α-angle, 1 to 3 patients for LI60 measurements and 1 to 4 patients for PT, APTT, fibrinogen or D-dimer.

Reference values

|  | CT (s) | CFT (s) | α (hoek) | A10 (mm) | MCF (mm) |
| --- | --- | --- | --- | --- | --- |
| INTEM | 100-240 | 30-110 | 70-83 | 48-69 | 50-72 |
| EXTEM | 38-79 | 34-159 | 63-83 | 48-69 | 50-72 |
| FIBTEM | - | - | - | 7-23 | - |

**Table S2B: Trajectory of ROTEM-parameters in aSAH patients with and without DCI, after exclusion of patients treated with tranexamic acid.**

|  | **T0** n=132 |  |  | **T1** n=100 |  |  | **T2** n=42 |  |  |
| --- | --- | --- | --- | --- | --- | --- | --- | --- | --- |
|  | **DCI +** (n=27) | **DCI -** (n=105) | **p-value** | **DCI +** (n=22) | **DCI -** (n=78) | **p-value** | **DCI +** (n=9) | **DCI -** (n=33) | **p-value** |
| **EXTEM CT** | 65 (62-73) | 64 (60-71) | 0.19 | 69 (60-71) | 63 (59-68) | 0.09 | 68 (65-84) | 66 (60-71) | 0.15 |
| **EXTEM CFT** | 55 (49-72) | 63 (54-76) | 0.08 | 48 (41-58) | 51 (42-65) | 0.25^†^ | 43 (36-65) | 46 (40-55) | 0.84 |
| **EXTEM α-angle** | **80 (78-82)** | **78 (76-81)** | **0.04** | 81 (80-82) | 81 (79-82) | 0.58^†^ | 81 (785-83) | 81 (79-82) | 0.91 |
| **EXTEM A10** | 60 (56-63) | 58 (55-61) | 0.42^†^ | 65 (61-67) | 62 (59-67) | 0.31^†^ | 68 (61-73) | 66 (63-70) | 1.00^†^ |
| **EXTEM MCF** | 69 (64-72) | 68 (65-70) | 0.17^†^ | 72 (69-75) | 70 (67-75) | 0.30^†^ | 78 (70-80) | 74 (71-77) | 0.65^†^ |
| **EXTEM LI60** | 99 (97-100) | 99 (97-99) | 0.48 | 97 (96-98) | 97 (95-98) | 0.92^†^ | 99 (99-100) | 98 (97-99) | 0.08 |
|  |  |  |  |  |  |  |  |  |  |
| **INTEM CT** | 150 (138-164) | 158 (141-174) | 0.32^†^ | 166 (153-174) | 167 (150-178) | 0.93^†^ | 159 (135-179) | 166 (154-179) | 0.81^†^ |
| **INTEM CFT** | 64 (52-81) | 69 (60-82) | 0.28^†^ | 56 (44-71) | 60 (47-73) | 0.51^†^ | 51 (36-69) | 47 (40-63) | 0.85^†^ |
| **INTEM α-angle** | 78 (75-80) | 77 (75-79) | 0.29^†^ | 80 (77-81) | 79 (77-81) | 0.32^†^ | 80 (77-83) | 81 (78-82) | 0.97^†^ |
| **INTEM A10** | 57 (53-60) | 55 (53-59) | 0.27^†^ | 62 (56-65) | 60 (56-64) | 0.72^†^ | 62 (59-70) | 64 (60-69) | 0.71^†^ |
| **INTEM MCF** | 65 (62-69) | 64 (61-67) | 0.13^†^ | 67 (65-72) | 67 (63-71) | 0.64^†^ | 74 (67-77) | 71 (67-75) | 0.69^†^ |
| **INTEM LI60** | 98 (96-100) | 98 (96-99) | 0.29 | 97 (94-98) | 97 (95-98) | 0.91^†^ | 99 (98-100) | 98 (96-99) | 0.06 |
|  |  |  |  |  |  |  |  |  |  |
| **FIBTEM α-angle** | 73 (64-76) | 72 (66-75) | 0.88^†^ | 79 (76-80) | 78 (74-80) | 0.17 | 78 (76-80) | 78 (76-80) | 0.72^†^ |
| **FIBTEM A10** | 15 (11-17) | 14 (12-16) | 0.44^†^ | 21 (20-26) | 20 (16-25) | 0.26^†^ | 22 (19-26) | 22 (20-27) | 0.76^†^ |
| **FIBTEM MCF** | 17 (13-18) | 15,5 (12-17) | 0.27^†^ | 24 (22-29) | 22 (18-27) | 0.24^†^ | 25 (21-30) | 25 (20-29) | 0.90^†^ |
|  |  |  |  |  |  |  |  |  |  |
| **PT** | 10.8 (10.6-11.2) | 11.0 (10.8-11.4) | 0.05 | 10.6 (10.1-10.8) | 10.6 (10.3-10.9) | 0.39 | 10.6 (10.2-11.0) | 10.7 (10.4-11.1) | 0.38 |
| **aPTT** | 22 (21-24) | 23 (22-25) | 0.12 | 22 (20-24) | 23 (21-25) | 0.10^†^ | 23 (21-24) | 22 (21-24) | 0.94 |
| **Fibrinogen** | 3,2 (2,8-3,5) | 3.1 (2.6-3.5) | 0.27^†^ | 5.3 (4.3-6.7) | 4.8 (3.9-6.3) | 0.26^†^ | 5.5 (4.3-7.1) | 5.7 (4.5-6.9) | 0.89^†^ |
| **D-dimer** | 2,3 (1,6-7.4) | 2.3 (1.0-5.2) | 0.36 | **1.4 (1.1-2.2)** | **1.0 (0.7-1.7)** | **0.04** | **1.8 (1.2-4.2)** | **1.1 (0.7-1.9)** | **0.03** |

All variables are displayed as median (IQR) regardless of the distribution of the data. Variables with normal distribution are, however, statistically tested using the T-test (indicated by †)
Data unavailable of 1 to 5 patients for MCF measurements, 2 to 5 patients for FIBTEM α-angle, 1 to 3 patients for LI60 measurements and 1 to 4 patients for PT, APTT, fibrinogen or D-dimer.

Reference values

|  | CT (s) | CFT (s) | α (hoek) | A10 (mm) | MCF (mm) |
| --- | --- | --- | --- | --- | --- |
| INTEM | 100-240 | 30-110 | 70-83 | 48-69 | 50-72 |
| EXTEM | 38-79 | 34-159 | 63-83 | 48-69 | 50-72 |
| FIBTEM | - | - | - | 7-23 | - |

|  | **T0** n=157 |  |  | **T1** n=125 |  |  | **T2** n=63 |  |  |
| --- | --- | --- | --- | --- | --- | --- | --- | --- | --- |
|  | **DCIrad +** (n=16) | **DCIrad -** (n=141) | **p-value** | **DCIrad +** (n=13) | **DCIrad -** (n=112) | **p-value** | **DCIrad +** (n=6) | **DCIrad -** (n=57) | **p-value** |
| **EXTEM CT** | 63 (60-69) | 64 (60-71) | 0.45^†^ | 68 (63-70) | 64 (59-70) | 0.23 | 67 (61-96) | 65 (61-69) | 0.19^†^ |
| **EXTEM CFT** | 55 (49-73) | 62 (54-74) | 0.22 | **45 (36-52)** | **52 (42-65)** | **0.03^†^** | 43 (37-55) | 47 (40-54) | 0.67^†^ |
| **EXTEM α-angle** | 80 (78-82) | 79 (77-81) | 0.07 | **82 (81-83)** | **81 (79-82)** | **0.04^†^** | 81 (79-83) | 81 (79-82) | 0.99^†^ |
| **EXTEM A10** | 62 (56-63) | 59 (56-62) | 0.19 | 66 (64-69) | 62 (59-67) | 0.06^†^ | 69 (65-72) | 66 (63-70) | 0.56^†^ |
| **EXTEM MCF** | 71 (65-73) | 68 (65-70) | 0.10 | **74 (71-76)** | **70 (68-74)** | **0.047^†^** | 77 (73-79) | 74 (71-77) | 0.27 |
| **EXTEM LI60** | 99 (98-100) | 99 (97-99) | 0.13 | 97 (96-99) | 97 (95-98) | 0.53 | 99 (97-100) | 98 (97-99) | 0.27 |
|  |  |  |  |  |  |  |  |  |  |
| **INTEM CT** | **138 (126-150)** | **157 (141-172)** | **0.002^†^** | 164 (153-175) | 167 (151-178) | 0.74^†^ | 170 (141-179) | 162 (151-177) | 0.78^†^ |
| **INTEM CFT** | 61 (52-70) | 67 (58-81) | 0.24 | 52 (39,5-64) | 61 (46-75) | 0.14^†^ | 46 (38-61) | 49 (40-61) | 0.77^†^ |
| **INTEM α-angle** | 78 (76-80) | 77 (75-79) | 0.36 | 80 (78-82) | 79 (77-81) | 0.17^†^ | 81 (79-83) | 80 (78-82) | 0.36^†^ |
| **INTEM A10** | 59 (53-60) | 56 (54-60) | 0.29 | 62 (57,5-65,5) | 60 (55-64) | 0.17^†^ | 66 (59-70) | 63 (61-69) | 0.96^†^ |
| **INTEM MCF** | 68 (61-69) | 64 (62-67) | 0.15^†^ | 70 (66,5-72) | 67 (63-71) | 0.12^†^ | 74 (70-76) | 71 (68-75) | 0.31 |
| **INTEM LI60** | 99 (97-100) | 98 (96-99) | 0.10 | 97 (94,5-99) | 97 (95-98) | 0.67^†^ | 100 (96-100) | 98 (96-99) | 0.24^†^ |
|  |  |  |  |  |  |  |  |  |  |
| **FIBTEM α-angle** | 75 (60-77) | 73 (67-75) | 0.55 | **80 (78-81)** | **77 (74-79)** | **0.02** | 80 (78-81) | 78 (76-80) | 0.18^†^ |
| **FIBTEM A10** | 16 (11-19) | 14 (12-16) | 0.57^†^ | **23 (20-29)** | **20 (16-24)** | **0.04^†^** | **27 (25-30)** | **21 (19-26)** | **0.03** |
| **FIBTEM MCF** | 17 (12-20) | 16 (13-18) | 0.56^†^ | **25 (23-33)** | **22 (18-27)** | **0.02^†^** | 30 (28-32) | 24 (20-29) | 0.06^†^ |
|  |  |  |  |  |  |  |  |  |  |
| **PT** | **10.8 (10.5-10.9)** | **11.0 (10.8-11.4)** | **<0.001** | **10.3 (9.8-10.6)** | **10.6 (10.4-10.9)** | **0.02** | 10.5 (10.2-10.7) | 10.7 (10.4-11.2) | 0.56^†^ |
| **aPTT** | **21 (20-23)** | **23 (22-25)** | **0.007** | 22 (20-23) | 23 (21-25) | 0.06 | 22 (20-23) | 22 (21-24) | 0.44^†^ |
| **Fibrinogen** | 3.3 (2.8-3.6) | 3.1 (2.7-3.5) | 0.60^†^ | **5.8 (4.7-7.9)** | **4.8 (4.1-6.2)** | **0.02^†^** | 6.5 (5.4-7.3) | 5.3 (4.4-6.4) | 0.14^†^ |
| **D-dimer** | 2.3 (1.3-11.8) | 1.9 (1.0-4.6 | 0.19^†^ | 1.2 (1.1-1.9) | 1.0 (0.7-1.7) | 0.25 | 2.3 (0.9-4.6) | 1.2 (0.8-2.0) | 0.27 |

**Table S3A: Trajectory of ROTEM-parameters in patients with and without radiological DCI.**

DCIrad: radiological DCI
All variables are displayed as median (IQR) irregardless of the distribution of the data. Variables with normal distribution are, however, statistically tested using the T-test (indicated by †)
Data unavailable of 1 to 5 patients for MCF measurements, 2 to 5 patients for FIBTEM α-angle, 1 to 3 patients for LI60 measurements and 1 to 4 patients for PT, APTT, fibrinogen or D-dimer.

Reference values

|  | CT (s) | CFT (s) | α (hoek) | A10 (mm) | MCF (mm) |
| --- | --- | --- | --- | --- | --- |
| INTEM | 100-240 | 30-110 | 70-83 | 48-69 | 50-72 |
| EXTEM | 38-79 | 34-159 | 63-83 | 48-69 | 50-72 |
| FIBTEM | - | - | - | 7-23 | - |

**Table S3B: Trajectory of ROTEM-parameters in patients with and without radiological DCI, after exclusion of patients treated with tranexamic acid.**

|  | **T0** n=132 |  |  | **T1** n=100 |  |  | **T2** n=42 |  |  |
| --- | --- | --- | --- | --- | --- | --- | --- | --- | --- |
|  | **DCIrad +** (n=13) | **DCIrad -** (n=119) | **p-value** | **DCIrad +** (n=10) | **DCIrad -** (n=90) | **p-value** | **DCIrad +** (n=3) | **DCIrad -** (n=39) | **p-value** |
| **EXTEM CT** | 63 (62-71) | 64 (60-71) | 1.00 | 69 (63-71) | 63 (59-69) | 0.09 | - | - | - |
| **EXTEM CFT** | 55 (49-72) | 62 (54-76) | 0.16 | 48 (42-52) | 51 (42-64) | 0.19^†^ | - | - | - |
| **EXTEM α-angle** | 80 (79-82) | 79 (76-81) | 0.06 | 82 (80-83) | 81 (79-82) | 0.21^†^ | - | - | - |
| **EXTEM A10** | 61 (57-63) | 58 (55-62) | 0.20^†^ | 65 (63-66) | 62 (59-67) | 0.29^†^ | - | - | - |
| **EXTEM MCF** | 71 (66-74) | 68 (65-70) | 0.08^†^ | 74 (70-76) | 70 (67-75) | 0.13^†^ | - | - | - |
| **EXTEM LI60** | 99 (99-100) | 99 (97-99) | 0.08 | 98 (96-99) | 97 (95-98) | 0.11^†^ | - | - | - |
|  |  |  |  |  |  |  |  |  |  |
| **INTEM CT** | **143 (122-151)** | **158 (144-174)** | **0.004^†^** | 169 (149-177) | 167 (152-178) | 0.86^†^ | - | - | - |
| **INTEM CFT** | 60 (52-77) | 69 (59-81) | 0.62^†^ | 61 (45-66) | 60 (46-74) | 0.54^†^ | - | - | - |
| **INTEM α-angle** | 78 (76-80) | 77 (75-79) | 0.54^†^ | 79 (78-81) | 79 (77-81) | 0.58^†^ | - | - | - |
| **INTEM A10** | 59 (54-61) | 55 (53-59) | 0.31^†^ | 61 (56-65) | 60 (56-64) | 0.53^†^ | - | - | - |
| **INTEM MCF** | 68 (62-70) | 64 (61-67) | 0.10^†^ | 69 (66-72) | 67 (63-71) | 0.28^†^ | - | - | - |
| **INTEM LI60** | 99 (98-100) | 98 (96-99) | 0.06 | 98 (95-99) | 97 (95-98) | 0.15^†^ | - | - | - |
|  |  |  |  |  |  |  |  |  |  |
| **FIBTEM α-angle** | 75 (62-77) | 72 (66-75) | 0.85 | 79 (78-80) | 78 (74-80) | 0.15 | - | - | - |
| **FIBTEM A10** | 16 (12-19) | 14 (11-16) | 0.34^†^ | 22 (20-29) | 20 (16-24) | 0.10^†^ | - | - | - |
| **FIBTEM MCF** | 17 (13-20) | 16 (13-17) | 0.36^†^ | 25 (22-33) | 22 (18-27) | 0.08^†^ | - | - | - |
|  |  |  |  |  |  |  |  |  |  |
| **PT** | **10.7 (10.6-10.9)** | **11.0 (10.8-11.4)** | **0.002** | 10.5 (9.7-10.6) | 10.6 (10.3-10.9) | 0.07 | - | - | - |
| **aPTT** | **21 (20-24)** | **23 (22-25)** | **0.04** | 22 (20-24) | 23 (21-25) | 0.19^†^ | - | - | - |
| **Fibrinogen** | 3.3 (2.9-3.6) | 3.1 (2.7-3.5) | 0.32^†^ | **5.4 (4.7-8.4)** | **4.7 (4.0-6.1)** | **0.03^†^** | - | - | - |
| **D-dimer** | 2.5 (2.0-17.1) | 2.2 (1.0-5.2) | 0.16 | 1.4 (1.1-2.1) | 1.0 (0.7-1.8) | 0.09 | - | - | - |

DCIrad: radiological DCI
All variables are displayed as median (IQR) irregardless of the distribution of the data. Variables with normal distribution are, however, statistically tested using the T-test (indicated by †)
Data unavailable of 1 to 5 patients for MCF measurements, 2 to 5 patients for FIBTEM α-angle, 1 to 3 patients for LI60 measurements and 1 to 4 patients for PT, APTT, fibrinogen or D-dimer.

Reference values

|  | CT (s) | CFT (s) | α (hoek) | A10 (mm) | MCF (mm) |
| --- | --- | --- | --- | --- | --- |
| INTEM | 100-240 | 30-110 | 70-83 | 48-69 | 50-72 |
| EXTEM | 38-79 | 34-159 | 63-83 | 48-69 | 50-72 |
| FIBTEM | - | - | - | 7-23 | - |

**Table S4 Frequency of common complications of aSAH in patients with good and poor clinical outome**

|  | **Good outcome (n=85)** | **Poor outcome (n=68)** | **P-value** |
| --- | --- | --- | --- |
| Rebleeding | 12 ((14) | 16 (24) | 0.15 |
| Hydrocephalus  CSF drainage  Lumbar puncture(s) | 51 (60)  51  13 | 55 (81)  54  32 | **0.01** |
| DCI | 18 (21) | 13 (19) | 0.84 |
| Treatment-related  Hemorrhagic complications  Thrombo-embolic complication  Cerebral ischemia | 4 (5)  3 (4)  2 (2) | 2 (3)  2 (3)  4 (6) | 1.00  1.00  0.19 |
| Meningitis | 2 (2) | 7 (10) | 0.08 |
| Pneumonia | 7 (8) | 14 (21) | **0.03** |
| Urinary tract infection | 8 (9) | 2 (3) | 0.19 |
| Seizures | 7 (8) | 15 (22) | **0.02** |

**Table S5A: Trajectory of ROTEM-parameters of patients with good and poor clinical outcome at six months post-aSAH**

|  | **T0** n=150 |  |  | **T1** n=120 |  |  | **T2** n=61 |  |  |
| --- | --- | --- | --- | --- | --- | --- | --- | --- | --- |
|  | **Good outcome** (n=83) | **Poor outcome-** (n=67) | **p-value** | **Good outcome** (n=79) | **Poor outcome-** (n=41) | **p-value** | **Good outcome** (n=43) | **Poor outcome-** (n=18) | **p-value** |
| **EXTEM CT** | **63 (58-69)** | **66 (61-73)** | **0.006** | 63 (59-70) | 65 (60-71) | 0.29 ^ttest^ | 64 (61-70) | 65 (61-68) | 0.81 |
| **EXTEM CFT** | 61 (54-72) | 61 (52-74) | 0.83 | **55 (47-65)** | **43 (36-70)** | **<0.001^†^** | **49 (43-58)** | **41 (37-43)** | **0.004** |
| **EXTEM α-angle** | 79 (76-81) | 79 (77-81) | 0.84 | **80 (79-82)** | **82 (80-83)** | **<0.001^†^** | **81 (79-82)** | **82 (81-82)** | **0.01** |
| **EXTEM A10** | 59 (55-62) | 60 (56-62) | 0.27^†^ | **61 (59-66)** | **67 (63-70)** | **<0.001^†^** | **65 (63-69)** | **69 (68-72)** | **0.01^†^** |
| **EXTEM MCF** | 68 (64-70) | 69 (66-71) | 0.13^†^ | **69 (66-73)** | **75 (72-76)** | **<0.001^†^** | **74 (71-77)** | **77 (75-78)** | **0.01^†^** |
| **EXTEM LI60** | 98 (97-99) | 99 (97-99) | 0.07 | 97 (95-98) | 97 (96-98) | 0.55^†^ | 98 (97-99) | 99 (97-100) | 0.20 |
|  |  |  |  |  |  |  |  |  |  |
| **INTEM CT** | 155 (138-173) | 153 (141-168) | 0.50^†^ | 165 (153-177) | 167 (147-178) | 0.51^†^ | 162 (148-177) | 164 (144-177) | 0.58^†^ |
| **INTEM CFT** | 68 (57-81) | 66 (56-79) | 0.36^†^ | **64 (55-76)** | **46 (39-60)** | **<0.001^†^** | **53 (41-63)** | **44 (36-49)** | **0.04^†^** |
| **INTEM α-angle** | 77 (75-79) | 78 (76-79) | 0.58^†^ | **78 (77-80)** | **81 (79-82)** | **<0.001^†^** | **80 (78-82)** | **81 (80-83)** | **0.02^†^** |
| **INTEM A10** | 56 (52-60) | 57 (54-60) | 0.34^†^ | **58 (55-62)** | **64 (60-68)** | **<0.001^†^** | **62 (60-67)** | **68 (65-71)** | **0.01^†^** |
| **INTEM MCF** | 64 (61-67) | 65 (62-68) | 0.11^†^ | **65 (62-69)** | **71 (67-74)** | **<0.001^†^** | **70 (67-74)** | **73 (72-77)** | **0.01^†^** |
| **INTEM LI60** | 98 (96-99) | 99 (96-100) | **0.14** | 97 (94-99) | 97 (95-99) | 0.62^†^ | 98 (96-99) | 98 (97-99) | 0.49 |
|  |  |  |  |  |  |  |  |  |  |
| **FIBTEM α-angle** | 74 (66-76) | 72 (67-75) | 0.45 | **77 (74-79)** | **80 (78-81)** | **<0.001^†^** | **78 (75-79)** | **80 (78-81)** | **<0.001^†^** |
| **FIBTEM A10** | 14 (11-17) | 15 (12-16) | 0.94^†^ | **18 (15-21)** | **26 (23-30)** | **<0.001^†^** | **20 (19-23)** | **27 (24-32)** | **<0.001^†^** |
| **FIBTEM MCF** | 16 (13-18) | 16 (13-18) | 0.67^†^ | **21 (17-24)** | **29 (25-33)** | **<0.001^†^** | **23 (20-26)** | **29 (26-33)** | **<0.001** |
|  |  |  |  |  |  |  |  |  |  |
| **PT** | 10.9 (10.8-11.4) | 11 (10.7-11.4) | 0.71 | **10.6 (10.4-10.9)** | **10.5 (10.0-10.8)** | **0.03^†^** | 10.6 (10.3-11.2) | 10.7 (10.5-11.2) | 0.72 |
| **aPTT** | 23 (21-24) | 23 (21-25) | 0.51 | 23 (21-24) | 22 (20-24) | 0.24^†^ | **23 (21-24)** | **21 (20-23)** | **0.004** |
| **Fibrinogen** | 3.1 (2.8-3.6) | 3.2 (2.5-3.5) | 0.17^†^ | **4.4 (3.8-5.0)** | **6.7 (5.3-7.8)** | **<0.001^†^** | **5.1 (4.4-6.2)** | **6.6 (5.3-7.1)** | **0.004^†^** |
| **D-dimer** | **1.62 (0.7-3.0)** | **3.4 (1.6-7.4)** | **<0.001** | **0.9 (0.6-1.4)** | **1.2 (0.9-2.1)** | **0.005** | **1.1 (0.7-1.4)** | **2.1 (1.2-5.1)** | **<0.001** |

All variables are displayed as median (IQR) regardless of the distribution of the data. Variables with normal distribution are, however, statistically tested using the T-test (indicated by †)
Data unavailable of 1 to 5 patients for MCF measurements, 1 to 6 patients for FIBTEM α-angle, 3 to 6 patients for LI60 measurements and 1 to 7 patients for PT, APTT, fibrinogen or D-dimer.

Reference values

|  | CT (s) | CFT (s) | α (hoek) | A10 (mm) | MCF (mm) |
| --- | --- | --- | --- | --- | --- |
| INTEM | 100-240 | 30-110 | 70-83 | 48-69 | 50-72 |
| EXTEM | 38-79 | 34-159 | 63-83 | 48-69 | 50-72 |
| FIBTEM | - | - | - | 7-23 | - |

**Table S5B: Trajectory of ROTEM-parameters of patients with good and poor clinical outcome at six months post-aSAH, after exclusion of patients treated with tranexamic acid.**

|  | **T0** n=126 |  |  | **T1** n=96 |  |  | **T2** n=41 |  |  |
| --- | --- | --- | --- | --- | --- | --- | --- | --- | --- |
|  | **Good outcome** (n=67) | **Poor outcome-** (n=59) | **p-value** | **Good outcome** (n=62) | **Poor outcome-** (n=34) | **p-value** | **Good outcome** (n=29) | **Poor outcome-** (n=12) | **p-value** |
| **EXTEM CT** | **63 (58-67)** | **67 (63-74)** | **<0.001** | 63 (59-69) | 66 (61-71) | 0.08 | 67 (62-72) | 66 (63-71) | 0.90 |
| **EXTEM CFT** | 60 (53-78) | 62 (54-75) | 0.80 | **54 (47-67)** | **43 (35-49)** | **<0.001^†^** | **47 (44-59)** | **40 (35-45)** | **0.01** |
| **EXTEM α-angle** | 79 (76-81) | 79 (77-81) | 0.76 | **81 (79-82)** | **82 (81-83)** | **<0.001^†^** | **81 (79-82)** | **82 (81-83)** | **0.02** |
| **EXTEM A10** | 59 (55-62) | 59 (56-62) | 0.41^†^ | **61 (57-65)** | **67 (63-71)** | **<0.001^†^** | **66 (63-69)** | **70 (67-75)** | **0.03^†^** |
| **EXTEM MCF** | 68 (64-70) | 68 (65-71) | 0.24^†^ | **69 (66-73)** | **75 (72-77)** | **<0.001^†^** | **74 (71-78)** | **77 (75-81)** | **0.04^†^** |
| **EXTEM LI60** | 98 (97-99) | 99 (97-100) | 0.09 | 97 (95-98) | 97 (96-98) | 0.51^†^ | 98 (97-99) | 99 (97-100) | 0.29 |
|  |  |  |  |  |  |  |  |  |  |
| **INTEM CT** | 155 (141-178) | 155 (144-169) | 0.53^†^ | 166 (153-177) | 168 (147-178) | 0.72^†^ | 161 (154-180) | 167 (144-176) | 0.68^†^ |
| **INTEM CFT** | 70 (55-81) | 66 (59-81) | 0.55^†^ | **64 (55-77)** | **47 (39-60)** | **<0.001^†^** | 53 (42-65) | 42 (33-48) | 0.06^†^ |
| **INTEM α-angle** | 77 (74-79) | 77 (76-79) | 0.74^†^ | **78 (77-80)** | **81 (79-82)** | **<0.001^†^** | **80 (77-82)** | **82 (80-83)** | **0.03^†^** |
| **INTEM A10** | 56 (52-60) | 56 (54-59) | 0.60^†^ | **58 (54-61)** | **64 (60-69)** | **<0.001^†^** | **62 (60-67)** | **70 (63-74)** | **0.03^†^** |
| **INTEM MCF** | 64 (61-67) | 65 (62-68) | 0.21^†^ | **65 (62-69)** | **71 (67-74)** | **<0.001^†^** | **70 (67-74)** | **75,5 (70-79)** | **0.03^†^** |
| **INTEM LI60** | 98 (96-99) | 99 (96-100) | 0.18 | 97 (94-98) | 97 (95-98) | 0.64^†^ | 98 (96-99) | 98 (97-99) | 0.98 |
|  |  |  |  |  |  |  |  |  |  |
| **FIBTEM α-angle** | 74 (64-76) | 71 (67-75) | 0.97^†^ | **77 (73-79)** | **80 (78-81)** | **<0.001** | **78 (76-79)** | **80 (78-81)** | **0.006^†^** |
| **FIBTEM A10** | 14 (11-17) | 14 (12-16) | 0.85^†^ | **19 (15-21)** | **27 (22-31)** | **<0.001^†^** | **20 (19-24)** | **27 (25-33)** | **<0.001^†^** |
| **FIBTEM MCF** | 16 (12-18) | 16 (13-17) | 0.89^†^ | **21 (17-24)** | **28 (25-35)** | **<0.001^†^** | **23 (20-26)** | **30 (26-35)** | **<0.001^†^** |
|  |  |  |  |  |  |  |  |  |  |
| **PT** | 10.9 (10.7-11.4) | 11.0 (10.7-11.4) | 0.28 | **10.6 (10.4-10.9)** | **10.5 (10.0-10.8)** | **0.02** | 10.6 (10.3-11.1) | 10.8 (10.6-11.3) | 0.25 |
| **aPTT** | 23 (21-25) | 23 (22-25) | 0.84 | 23 (21-25) | 22 (21-24) | 0.32^†^ | **23 (21-25)** | **22 (20-23)** | **0.02** |
| **Fibrinogen** | 3.1 (2.7-3.5) | 3.2 (2.4-3.5) | 0.22^†^ | **4.4 (3.8-5.0)** | **6.8 (5.5-7.9)** | **<0.001^†^** | **4.8 (4.4-6.2)** | **6.9 (5.8-7.1)** | **0.004^†^** |
| **D-dimer** | **1.7 (0.8-3.4)** | **3.6 (1.8-9.6)** | **<0.001** | **1.0 (0.6-1.6)** | **1.4 (0.9-2.2)** | **0.03** | **1.1 (0.7-1.6)** | **2.7 (1.2-5.5)** | **0.002** |

All variables are displayed as median (IQR) regardless of the distribution of the data. Variables with normal distribution are, however, statistically tested using the T-test (indicated by †)
Data unavailable of 1 to 5 patients for MCF measurements, 1 to 6 patients for FIBTEM α-angle, 3 to 6 patients for LI60 measurements and 1 to 7 patients for PT, APTT, fibrinogen or D-dimer.

Reference values

|  | CT (s) | CFT (s) | α (hoek) | A10 (mm) | MCF (mm) |
| --- | --- | --- | --- | --- | --- |
| INTEM | 100-240 | 30-110 | 70-83 | 48-69 | 50-72 |
| EXTEM | 38-79 | 34-159 | 63-83 | 48-69 | 50-72 |
| FIBTEM | - | - | - | 7-23 | - |

**Figure S1: Medians (IQR) of ROTEM-parameters in patients with and without DCI, measured on admission, days 3-5 and days 9-11 after aSAH.**


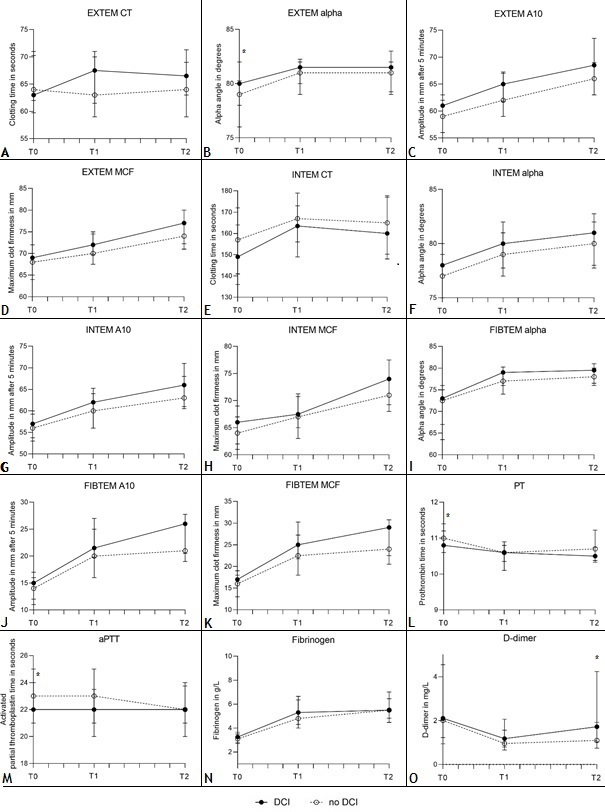


Median and IQR of (A) EXTEM CT, (B) EXTEM α-angle, (C) EXTEM A10, (D) EXTEM MCF, (E) INTEM CT, (F) INTEM α-angle, (G) INTEM A10, (H) INTEM MCF, (I) FIBTEM α-angle, (J) FIBTEM A10,(K) FIBTEM MCF, (L) PT, (M) aPTT, (N) fibrinogen, (O) D-dimer.

**Figure S2: ROC-curves on radiological DCI of ROTEM-parameters with a significant association with radiological DCI in univariate logistic regression analyses.**


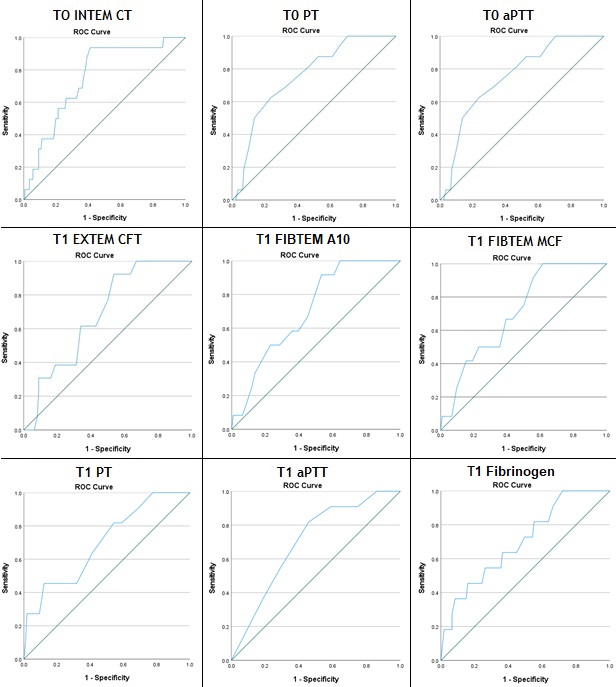


ROC-curve of (A)T0 INTEM CT, (B) T0 PT, (C) T0 aPTT, (D) T1 EXTEM CFT, (E) T1 FIBTEM A10, (F) T1 FIBTEM MCF, (G) T1 PT, (H) T1 aPTT, (I) T1 fibrinogen.

**Figure S3: ROC-curves on poor clinical outcome (mRS 4-6) of ROTEM-parameters with
a significant association with poor clinical outcome in univariate logistic regression analyses.**


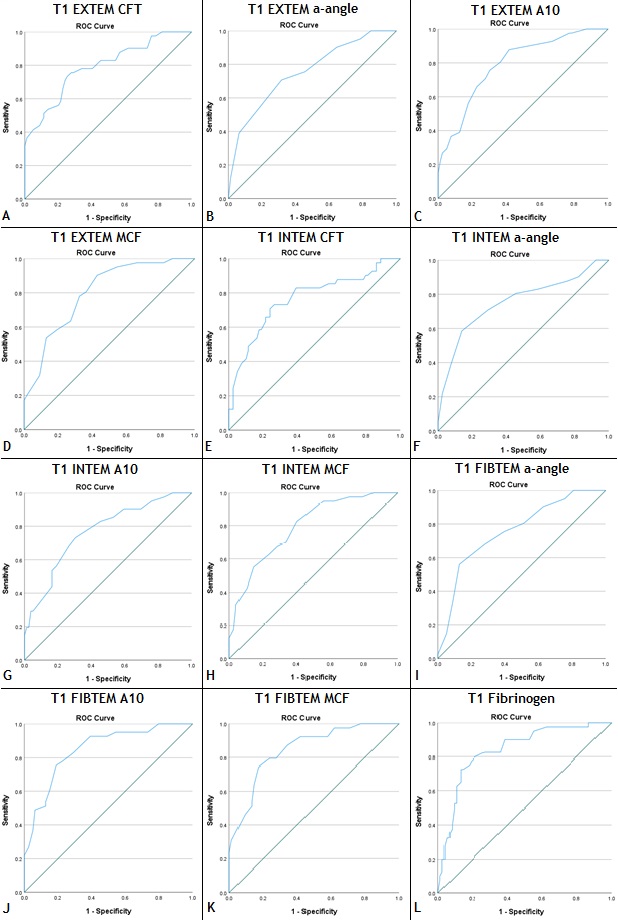


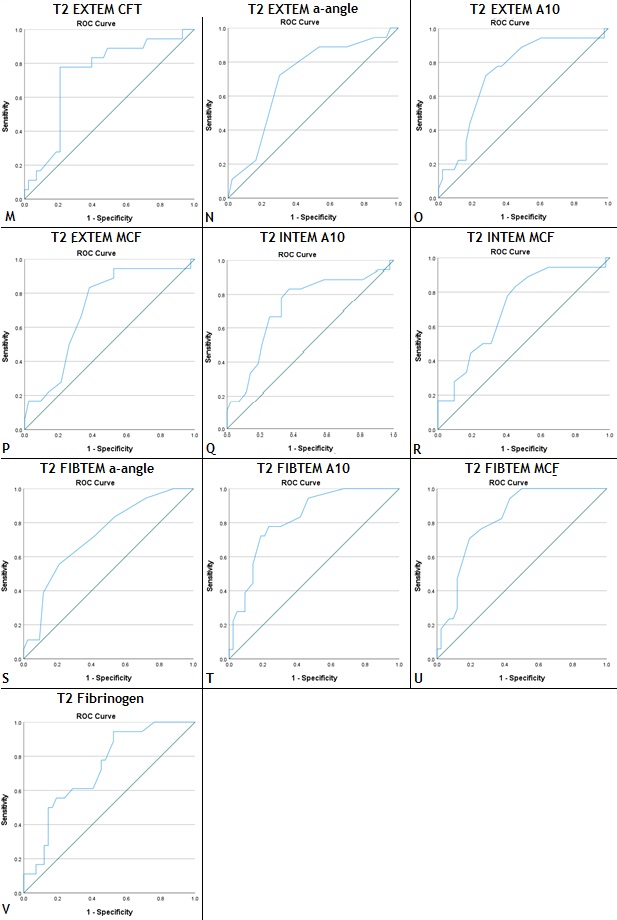


ROC-curve of (A) T1 EXTEM CFT, (B) T1 EXTEM α-angle, (C) T1 EXTEM A10, (D) T1 EXTEM MCF, (E) T1 INTEM CFT, (F) T1 INTEM α-angle, (G) T1 INTEM A10, (H) T1 INTEM MCF, (I) T1 FIBTEM α-angle, (J) T1 FIBTEM A10,(K) T1 FIBTEM MCF, (L) T1 fibrinogen, (M) T2 EXTEM CFT, (N) T2 EXTEM α-angle, (O) T2 EXTEM A10, (P) T2 EXTEM MCF, (Q) T2 INTEM A10, (R) T2 INTEM MCF, (S) TT2 FIBTEM α-angle, (T) T2 FIBTEM A10, (U) T2 FIBTEM MCF, (V) T2 fibrinogen
